# *Plasmodium* male gametocyte development and transmission are critically regulated by the two putative deadenylases of the CAF1/CCR4/NOT complex

**DOI:** 10.1371/journal.ppat.1007164

**Published:** 2019-01-31

**Authors:** Kevin J. Hart, Jenna Oberstaller, Michael P. Walker, Allen M. Minns, Mark F. Kennedy, Ian Padykula, John H. Adams, Scott E. Lindner

**Affiliations:** 1 Department of Biochemistry and Molecular Biology, Center for Malaria Research, Pennsylvania State University, University Park, State College, Pennsylvania, United States of America; 2 Center for Global Health and Infectious Diseases Research, Department of Global Health, University of South Florida, Tampa, Florida, United States of America; Umea Universitet, SWEDEN

## Abstract

With relatively few known specific transcription factors to control the abundance of specific mRNAs, *Plasmodium* parasites may rely more on the regulation of transcript stability and turnover to provide sufficient gene regulation. *Plasmodium* transmission stages impose translational repression on specific transcripts in part to accomplish this. However, few proteins are known to participate in this process, and those that are characterized primarily affect female gametocytes. We have identified and characterized *Plasmodium yoelii* (Py) CCR4-1, a putative deadenylase, which plays a role in the development and activation of male gametocytes, regulates the abundance of specific mRNAs in gametocytes, and ultimately increases the efficiency of host-to-vector transmission. We find that when *pyccr4-1* is deleted or its protein made catalytically inactive, there is a loss in the initial coordination of male gametocyte maturation and a reduction of parasite infectivity of the mosquito. Expression of only the N-terminal CAF1 domain of the essential CAF1 deadenylase leads to a similar phenotype. Comparative RNA-seq revealed that PyCCR4-1 affects transcripts important for transmission-related functions that are associated with male or female gametocytes, some of which directly associate with the immunoprecipitated complex. Finally, circular RT-PCR of one of the bound, dysregulated transcripts showed that deletion of the *pyccr4-1* gene does not result in gross changes to its UTR or poly(A) tail length. We conclude that the two putative deadenylases of the CAF1/CCR4/NOT complex play critical and intertwined roles in gametocyte maturation and transmission.

## Author summary

Malaria is a disease caused by *Plasmodium* parasites, which are transmitted during an infectious blood meal by anopheline mosquitoes. Transmission of the sexual stages of the parasite to mosquitoes requires the proper regulation of specific mRNAs. While much work has been done to characterize regulation of mRNAs in female gametocytes, little has been done to assess this regulation in male gametocytes. Here, we demonstrate that PyCCR4-1, a member of the CAF1/CCR4/NOT RNA metabolic complex, acts upon transcripts both directly and indirectly in gametocytes, and results in a reduction of male gametocytemia. In gametocytes lacking PyCCR4-1, as well as those expressing a catalytically dead variant, the initial coordinated wave of male gametocyte activation is lost, and these parasites are ~4-fold less able to productively infect mosquitoes. We find that the deletion of the C-terminal portion of CAF1 in both *Plasmodium yoelii* and *Plasmodium falciparum* phenocopies the deletion of *pyccr4-1*. We also find that the CAF1/CCR4/NOT complex is directly binding some of these transcripts and is likely acting both directly upon mRNAs and indirectly to modulate transcript abundance. These findings demonstrate that the combined effects of the CAF1/CCR4/NOT complex upon specific mRNAs are important for both male and female gametocytes, and that this regulation is required for efficient transmission to the mosquito vector.

## Introduction

Malaria remains one of the great global health problems today, with 216 million new infections and 445,000 deaths attributed to it annually [1]. Resistance to frontline drugs is spreading, and understanding the development and transmission of the malaria parasite is important to bolster efforts to reduce or eliminate deaths due to this infection. For the parasite to transmit from a vertebrate host to the mosquito vector, a small percentage of the cells will differentiate from asexual forms and develop into sexual stage gametocytes, which can persist in an infectious state until a mosquito takes a blood meal. This event allows a small number of gametocytes to be taken up into the mosquito, but with far fewer parasites productively infecting it [2]. Following two weeks of development within the mosquito, a small number of sporozoites will similarly be injected into a host by the mosquito as it takes another blood meal [3]. In the effort to develop vaccines and drugs, transmission events have been identified as prime targets to prevent the spread of the parasite because they are population bottlenecks in the parasite life cycle. We and others have focused upon the transmitted gametocyte and sporozoite stages of *Plasmodium* parasites to identify and exploit their weaknesses. In both cases, very few parasites are transmitted, and thus these bottlenecks are excellent points of intervention. The identification of molecular processes that are important for the transmission of the parasite in one or both of these events, and their modes-of-action, are thus top priorities for the development of new therapeutics.

Recent work has shown that the parasite requires tight transcriptional and translational control to navigate its complex transmission events [4–8]. Despite these strict requirements for the effective transmission of the parasite, *Plasmodium* has only one known, expanded family of specific transcription factors, the ApiAP2 proteins (27 members, reviewed in [9]). However, *Plasmodium* expresses a larger number of RNA-binding proteins than other eukaryotes (~10% of its proteome), which likely provides expanded capabilities to regulate gene expression post-transcriptionally to control translation, and to stabilize or degrade RNA [5, 10, 11]. For instance, *Plasmodium* uses RNA-binding proteins such as DOZI (an orthologue of the human DDX6 RNA helicase), CITH (an orthologue of Lsm14A), and ALBA family proteins to impose translational repression and mRNA stabilization on transcripts in female gametocytes that are important for the establishment of a new infection of a mosquito [10, 12–16]. A current model invokes these controls as a means for the parasite to always be ready to respond to external stimuli that indicate that transmission has occurred, and thus enables the “just-in-time” translation of the preserved mRNAs and establishment of the new infection [17]. Moreover, this molecular process is essential to the transmission of the parasite, as deletion of *dozi* or *cith* results in a complete arrest of development in early mosquito stage [14, 15]. Similar regulatory events occur in the other transmitted stage (sporozoites) via the PUF2 RNA-binding protein, as deletion of *puf2* results in the gradual loss of infectivity and subsequent premature dedifferentiation into a liver stage-like form while in the salivary gland [6–8]. As in model eukaryotes (*e*.*g*. yeast, flies, worms) and in humans, many of these regulatory functions of RNA metabolism occur in cytosolic granules within the parasite as well [14, 15, 18, 19].

In addition to transcript stabilization, translational control can also be accomplished by the degradation of transcripts. Degradation of mRNAs is typically initiated by deadenylases, which remove the protective poly(A) tail. Historically, it was concluded that shortening the poly(A) tail to a critical length in turn promotes the subsequent decapping and complete degradation of the transcript by other factors [20, 21]. However, recent work has shown that mRNAs with short poly(A) tails can remain stable and are actively translated [22]. In many eukaryotes, the main complex responsible for deadenylation is the CAF1/CCR4/NOT complex, which also participates in transcriptional elongation, translational repression, and histone modification functions, and thus acts broadly upon gene expression [21]. This complex typically contains two putative deadenylases, with CAF1 (CCR4-Associated Factor 1, POP2) serving as the major deadenylase and CCR4 (Carbon Catabolite Repressor 1) playing additional or specialized roles, except for in yeast where the roles are reversed [21]. While CAF1’s role in binding to and degrading poly(A) tracts is best appreciated, it has been shown to bind several other poly-nucleotide tracts [23]. Recently, a cryo EM structure of the *S*. *pombe* CAF1/CCR4/NOT complex was reconstructed using immunoprecipitated material. This work confirmed previous studies that used recombinant proteins and binding assays to show that the complex is L-shaped, that NOT1 (Negative on TATA-less 1) acts as the scaffold, and that CCR4 binds to the complex indirectly through bridging interactions with CAF1 [24, 25]. While these associations and activities have been well described in many eukaryotes, little is known about the CAF1/CCR4/NOT complex’s form and function in malaria parasites.

In *Plasmodium*, previous work confirmed that normal deadenylase activity provided by CAF1 is essential for asexual blood stage growth [26, 27]. Interestingly, insertion of a *piggyBac* transposon into the coding sequence revealed that CAF1 contributes to the regulation of invasion and egress-related genes in asexual blood stage parasites [26]. It is possible that this transposon insertion still results in the production of a partially functional CAF1 protein. Multiple independent attempts to knock out *caf1* in the rodent-infectious species *P*. *berghei* failed, indirectly indicating that it is essential for parasite development [26, 28]. Moreover, previous work on *Plasmodium* sporozoites identified that deletion of *pypuf2* led to significant changes in the transcript abundance of several members of the CAF1/CCR4/NOT complex [6, 29]. Among the affected transcripts, two mRNAs encoding CCR4 domain-containing proteins were dysregulated. As the deadenylase proteins of the CAF1/CCR4/NOT complex have been shown to be specialized regulators in other species, we investigated the possibility that CCR4 domain-containing proteins may be acting in this capacity in *Plasmodium* as well [30, 31].

Here, we demonstrate that CCR4-1 is a specialized regulator during gametocytogenesis and transmission of the rodent-infectious *Plasmodium yoelii* parasite from the mammalian host to the mosquito vector. Deletion of *pyccr4-1*, or expression of a putatively catalytic dead variant, resulted in a loss of the initial synchronous development of male gametocytes that can activate into gametes, as well as a reduction in the total number of mature male gametocytes. Moreover, deletion of *pyccr4-1* also reduced the transmissibility of the parasite to the mosquito on both peak and post-peak transmission days, indicating that PyCCR4-1’s functions extend beyond its role in the partial synchronization of gametocytes. Comparative transcriptomics of wild-type and *ccr4-1*^*-*^ gametocytes revealed that PyCCR4-1 significantly impacts the abundance of transcripts that are translationally repressed in female gametocytes, and those that impact the transmission to and establishment of an infection in the mosquito. We found that PyCCR4-1 binds directly to some of these affected transcripts and allows for increased transcript abundance without affecting UTR or poly(A) tail length. Surprisingly, this effect runs counter to the major canonical role of a deadenylase. Finally, proteomic characterizations and genetic modifications of *pycaf1* and *pfcaf1* indicate that the C-terminal region of CAF1 is needed for proper gametocyte development and to promote host-to-vector transmission.

## Results

### PyCCR4-1 is important for the development and transmission of male gametocytes

To first assess the importance of the CAF1/CCR4/NOT complex in *Plasmodium*, we bioinformatically identified the genes for all members of the canonical CAF1/CCR4/NOT complex in *Plasmodium*, except for *not3* and *caf130*. The absence of these two particular genes is not surprising, as these genes are also absent in some eukaryotes [20]. In addition, we identified four CCR4 domain-containing proteins (PyCCR4-1, PyCCR4-2, PyCCR4-3, PyCCR4-4) that have homology to CCR4 deadenylases in other eukaryotes (*e*.*g*. yeast, human, mouse) (S1A and S1B Fig) through BLASTp alignments [32]. The typical domain architecture of CCR4-like proteins involves a Leucine Rich Repeat Region (LRR) and an Endonuclease/Exonuclease/Phosphatase (EEP) domain. The LRR mediates the interaction of CCR4 with CAF1 and the rest of the NOT complex, while the EEP domain contains active site residues required for deadenylation activity. Of these, we found that the overall length and sequence conservation within the EEP domain of PyCCR4-1 aligns most closely with the consensus CCR4 domain-containing proteins from model eukaryotes and humans (S1A Fig). However, beyond the CCR4-EEP domain, there is no significant homology between other regions from PyCCR4-1, 2, 3, and 4 to each other, or to homologues from model species (S1B Fig) [33].

**Fig 1:**
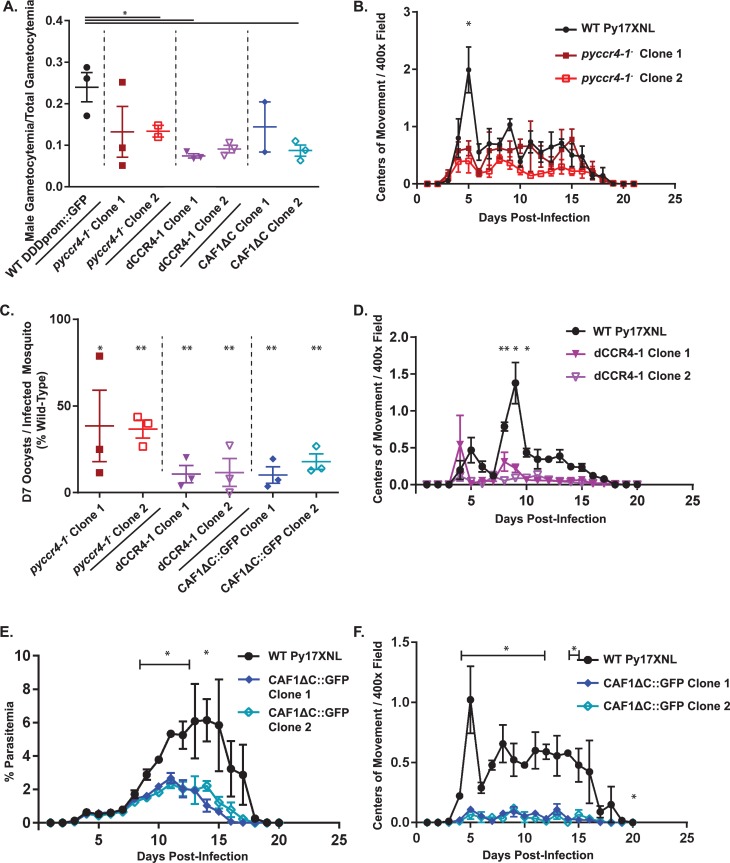
In *pyccr4-1*^*-*^, dCCR4-1, and PyCAF1ΔC parasites, a semi-synchronous coordination of gametocyte maturation is lost and parasite transmissibility is reduced. A) The number of mature male gametocytes was determined by flow cytometry of sulfadiazine-treated and DDD/BIP-stained *P*. *yoelii* parasites and is plotted as a fraction of total gametocytes. * denotes a p-value <0.05. B,D,F) The number of centers-of-movement/exflagellation centers were quantified daily by microscopy on a 400x field of RBCs taken from mice infected with 10,000 (B, F) or 1,000 (D) blood stage parasites. Fewer wild-type and dCCR4-1 parasites were injected to better preserve animal health over the course of the experiment in accordance with our IACUC protocol (D). Plotted are three biological replicates with three technical replicates each. Error bars represent the standard error of the mean. C) The number of oocysts per infected mosquito on day seven post-infectious blood meal are plotted. Data represents at least 20 dissected mosquitoes per biological replicate conducted in triplicate. Error bars represent the standard error of the mean. E) Parasitemia was measured microscopically by Giemsa-stained thin blood smears. Plotted are three biological replicates with three technical replicates each. Error bars represent the standard error of the mean.

As deadenylases are also known to act as translational regulators in specific and temporal manners, we investigated the role of all four CCR4 domain-containing proteins throughout the *Plasmodium* life cycle [34, 35]. Our recent RNA-sequencing data from *Plasmodium yoelii* shows that all four genes are expressed in asexual blood stages and in gametocytes [4], and thus we sought to determine if any of the CCR4 domain-containing proteins played an important, stage-specific role in the parasite life cycle. To this end, we replaced the coding sequences of *pyccr4-1*, *pyccr4-2*, *pyccr4-3*, *pyccr4-4* with a GFP-expression cassette and a human dihydrofolate reductase (HsDHFR)-expression cassette via double homologous recombination in the *Plasmodium yoelii* 17XNL strain (S1C, S1D, S1E and S1F Fig). These lines were cloned via limiting dilution prior to characterization and their transgenic genotypes were confirmed using PCR across both homology regions. These clonal parasites revealed that deletion of any one of these genes individually was not lethal in asexual blood stages. Moreover, deletion of *pyccr4-2*, *pyccr4-3*, or *pyccr4-4* resulted in transgenic parasites that behaved as wild-type in all life cycle stages with respect to parasite numbers, prevalence of mosquito infection, and developmental timing/completion throughout the *Plasmodium* life cycle (Fig 1A, 1B and 1C, S1 Table). Thus, CCR4-2, -3, and -4 may play redundant roles with one or more of the other CCR4-domain containing proteins.

However, while deletion of *pyccr4-1* had no effect upon asexual blood stage growth (S2A Fig), it led to significant phenotypes during male gametocyte maturation and host-to-vector transmission (Fig 1A, 1B and 1C). First, to assess gametocytogenesis and the number of mature male gametocytes present, we developed an antibody-based flow cytometry assay based in part upon the effective reporter system (820cl1m1cl1) commonly used in *P*. *berghei* [15]. We generated antibodies against a recombinant domain variant of dynein heavy chain delta (PyDD, PY17X_0418900, “PyDDD” = AA1845-2334), and together with anti-PvBiP antibodies to counterstain cells containing a parasite, we confirmed by flow cytometry and Giemsa staining that PyDD is a marker for mature male gametocytes in *P*. *yoelii*, as it is in *P*. *berghei* (S2B Fig). This was further validated using a transgenic parasite line with a PyDDprom::GFPmut2 cassette integrated in the *p230p* safe harbor locus, where the population positive for both anti-PyDD and anti-GFP signals highly overlapped (S2B Fig). The *p230p* safe harbor locus has been used extensively when expression out of a dispensable locus is required [4, 15]. Ultimately, this approach allows these measurements to be done without the need to conduct reverse genetics in a base fluorescent reporter line, and frees up the use of GFP and RFP for other purposes.

Using this flow cytometric method, we found that transgenic *pyccr4-1*^*-*^ parasites produce fewer mature male gametocytes and more immature/female gametocytes compared to wild-type parasites (Fig 1A, S2C Fig). Secondly, in contrast to wild-type parasites, which have a semi-synchronous wave of gametocyte development, *pyccr4-1*^*-*^ parasites lose this coordination and instead develop fewer male gametocytes that can form gametes, and do so in an asynchronous manner (Fig 1B).

### PyCCR4-1 associates with a canonical CAF1/CCR4/NOT complex

Total proteomics of mixed blood stage samples of *P*. *yoelii* wild-type and *pyccr4-1*^*-*^ parasite lines indicated that many of the bioinformatically identified members of this complex (NOT1, NOT4, NOT5, NOT Family Protein, CAF40) are expressed at levels that permit their detection, whereas CCR4-1 and CAF1 were not sufficiently abundant to be detected using highly stringent thresholds (S2 Table).

To experimentally determine the composition of this complex in *Plasmodium yoelii*, a transgenic PyCCR4-1::GFP parasite was created (S2D Fig). The CAF1/CCR4/NOT complex was immunoprecipitated via the GFP tag from synchronized schizonts, when PyCCR4-1 is most abundant and is most prominently localized to cytoplasmic granules (S2D Fig). As seen in other eukaryotes, PyCCR4-1 associates directly or indirectly through bridging interactions with most members of the canonical CAF1/CCR4/NOT complex in *P*. *yoelii* (Table 1, S3 Table) [21]. Specifically, through mass spectrometric analyses we found that PyCCR4-1 associates with CAF1, NOT1, CAF40, NOT2 and a NOT family protein above our most stringent SAINT (Significance Analysis of INTeractome) threshold (0.1), and with NOT5 using a less stringent threshold (0.1 to 0.35). A small number of peptide spectral matches for NOT4 were also observed, but were not sufficiently enriched to be confidently included. This low abundance of NOT4 is consistent with its known transient association with the CAF1/CCR4/NOT complex in other eukaryotes [36]. We also found that PyCCR4-1 interacts with proteins involved in the nuclear pore complex and RNA export (*e*.*g*. karyopherin-beta 3, exportin-1, UAP56), proteins involved in translation initiation (*e*.*g*. eIF2A, EF-1, EIF3D, PABP), and translational repression (*e*.*g*. CELF2/Bruno, DOZI, CITH, PABP) (Table 1, S3 Table) [21]. All of these interactions are consistent with appreciated CAF1/CCR4/NOT protein-protein or protein-RNA-protein interactions in other eukaryotes. Recently, a proteome of stress granule components in *S*. *cerevisiae* defined several cytosolic granule regulators, several of which we also found associated with PyCCR4-1 [37]. Specifically, we identified that multiple CCT proteins of the TRiC complex (*e*.*g*. CCT4, CCT5, CCT8) and HSP40-A associate (SAINT score < 0.1), and this list expands to include the remainder of the TRiC core complex (*e*.*g*. TCP1, CCT2, CCT3, CCT6, CCT7) and a regulatory kinase (CK1) (SAINT scores between 0.1 to 0.35). CCT proteins are known to inhibit stress granule formation in yeast and may be present with the CAF1/CCR4/NOT complex while it plays roles in active translation [37]. In addition, recent work has implicated karyopherins/nuclear import receptors in the regulation of proteins found within liquid-liquid phase separations/cytosolic granules [38]. Here, we identified that karyopherin beta 3 associates with the *P*. *yoelii* CAF1/CCR4/NOT complex, and perhaps indicates that similar regulatory processes are at work. These data indicate that the composition of the CAF1/CCR4/NOT complex, including the presence of cytosolic granule regulators, are likely conserved throughout eukaryotes, including *Plasmodium*.

**Table 1:** A list of proteins that interact with PyCCR4-1::GFP. Shading of cells indicates stringency thresholds (unshaded = highest, light gray = high, dark gray low).

		PyCCR4-1::GFP		
Gene ID	Name	Spectra	Control Spectra	SAINT Score
CAF1/CCR4/NOT Complex				
PY17X_1237700	CCR4-1	127|112|22	N/A	N/A
PY17X_1027900	NOT family protein putative	97|132|19	0|0|0	0
PY17X_0945600	NOT1	50|46|7	0|0|0	0
PY17X_1428300	CAF1	47|58|11	0|0|0	0
PY17X_1108300	CAF40	17|20|7	0|0|0	0.0004
PY17X_0921700	NOT2	8|12|3	0|0|0	0.002
PY17X_1207500	NOT5	7|2|0	0|0|0	0.2196
PY17X_1452400	NOT4	0|1|0	0|0|0	0.8736
Translational Repressors				
PY17X_1220900	DOZI	12|13|4	0|0|0	0.0011
PY17X_1035100	CELF2	17|21|5	1|0|0	0.0037
Stress Granule				
PY17X_0311400	CCT8	10|3|3	0|0|0	0.0131
PY17X_0613400	HSP40 subfamily A	15|15|5	0|3|0	0.0368
PY17X_1135600	CCT4	12|5|1	1|0|0	0.0735
PY17X_0913800	TSN	7|10|1	1|0|0	0.0774
PY17X_1221400	CCT5	4|2|1	0|0|0	0.0847
PY17X_0822200	HSP70-2	17|8|5	3|0|0	0.0882
Nuclear Transport				
PY17X_0307400	UAP56	9|4|6	0|0|0	0.0071
PY17X_0403700	exportin-1	8|12|2	0|1|0	0.033
PY17X_1242000	karyopherin beta-3	39|64|16	6|6|0	0.0525
PY17X_1416100	exportin-1	3|4|2	0|0|0	0.0694
Translation				
PY17X_1209300	EIF3D	9|9|3	0|0|0	0.0028
PY17X_0926900	EF-1 subunit alpha	4|6|3	0|0|0	0.0205
PY17X_0504300	eIF2A	15|33|2	0|0|0	0.026
PY17X_0309700	peptide chain release factor subunit 1	3|2|2	0|0|0	0.057
PY17X_1034300	eIF2 gamma	3|3|1	0|0|0	0.0612
PY17X_1369900	60S ribosomal protein L17	6|6|2	1|0|0	0.0653
Other				
PY17X_0933200	HSP101	15|13|10	0|0|0	0.0002
PY17X_0104900	putative anonymous antigen-1*	17|19|3	0|0|0	0.0044
PY17X_0814600	Ran-binding protein	8|7|4	0|0|0	0.0058
PY17X_1402200	cytoadherence linked asexual protein	12|8|3	1|0|0	0.0155
PY17X_0109000	OAT	5|4|3	0|0|0	0.0131
PY17X_1139200	glideosome-associated connector*	34|30|5	0|4|0	0.0181
PY17X_1016400	coatamer protein beta subunit	5|9|2	0|0|0	0.0226
PY17X_1322200	6-phosphogluconate dehydrogenase decarboxylating	6|8|1	0|0|0	0.0294
PY17X_1313800	M17 leucyl aminopeptidase putative	7|2|2	0|0|0	0.0405
PY17X_0833500	RhopH2	18|12|13	1|3|0	0.0441
PY17X_0418800	RhopH3	20|9|6	3|0|0	0.0479
PY17X_0708300	SEC23	2|6|2	0|0|0	0.0811
PY17X_0525700	tryptophan/threonine-rich antigen	2|2|2	0|0|0	0.0915
PY17X_1411400	meiosis-specific nuclear structural protein 1	2|2|3	0|0|0	0.0949
PY17X_0621600	putative hydrolase/p36 like protein*	5|1|1	0|0|0	0.0988

### The putative catalytic residues of PyCCR4-1 are required for its roles in gametocytogenesis and transmission

CCR4 proteins have well defined, conserved catalytic residues in other eukaryotes that are also conserved in *Plasmodium* species (S1B Fig) [39]. To determine if the putative active site residues of PyCCR4-1 contribute to its functions in male gametocyte maturation and transmission, we created transgenic parasites with alanine substituted for two of the putative catalytic residues (D1852A, H1898A) of PyCCR4-1 (dCCR4-1) (S1B Fig, S2E Fig). Like *pyccr4-1*^*-*^ parasites, dCCR4-1 transgenic parasites also produce fewer mature male gametocytes, and also lacked a synchronous wave of male activation (Fig 1A and 1D).

Because some male gametocytes retained the ability to mature and become exflagellating gametes in both the *pyccr4-1*^*-*^ and dCCR4-1 lines, we assessed whether they were transmissible to mosquitoes. In both transgenic lines, we observed a corresponding decrease of similar scale (2-to-10 fold) in the number of day seven oocysts compared to wild-type parasites when transmitted to *An*. *stephensi* on the peak day of male gametocyte activation into gametes (Fig 1C). Moreover, although there is no statistical difference in the number of male gametocytes that can activate between wild-type and *pyccr4-1*^*-*^ parasites after the peak day (Fig 1B, days six and beyond), a significant decrease (~30% of wild-type, p<0.05) in the number of oocysts in the mosquito was still observed when parasites were transmitted two days post-peak (S2F Fig). These data indicate that the catalytic residues of PyCCR4-1 are required for normal male gametocyte development and host-to-vector transmission.

### Truncation of PyCAF1 phenocopies the deletion of *pyccr4-1*

We next sought to determine if the other deadenylase in the CAF1/CCR4/NOT complex, CAF1, was required for these effects upon gametocyte development and host-to-vector transmission [40–42]. As CCR4 domain-containing proteins associate with the NOT1 scaffold of the CAF1/CCR4/NOT complex indirectly by binding CAF1, genetic deletion of *caf1* would theoretically dissociate CCR4-1 from its complex. However, complete deletions of the *caf1* gene have been unsuccessful in both a conventional targeted attempt and in the PlasmoGEM broad-scale genetic screen in *P*. *berghei*, indicating that it is likely essential [26, 43]. We also attempted to completely delete the *P*. *yoelii caf1* coding sequence and similarly were unable to delete these sequences (S3A Fig). Instead, as the insertion of the *piggyBac* transposon into the *P*. *falciparum caf1* gene occurred in the coding sequence downstream of the CAF1 domain, we hypothesized that this 5’ portion of *pfcaf1* mRNA may still be expressed and may encode the necessary portion of the protein [26]. In support of this hypothesis, transcript expression analysis of the *P*. *falciparum* CAF1 disruptant line (PfCAF1ΔC) bearing this transposon insertion indicated that the CAF1 domain was still transcribed up to the insertion site, but not after (S3B Fig).

Based upon these expression data, we created a *Plasmodium yoelii* transgenic line that mimics this transposon insertion by inserting a C-terminal GFP tag and stop codon in the *Plasmodium yoelii caf1* gene in a comparable location following the CAF1 domain, thus creating a PyCAF1ΔC (AA 1–335) variant (S3C Fig) [26]. We found that expression of the PyCAF1ΔC::GFP variant resulted in viable parasites, but importantly, that these parasites exhibit a similar growth attenuation as was observed for the *P*. *falciparum* PfCAF1ΔC line (Fig 1E) [26]. To further assess the impact of the PyCAF1ΔC variant upon parasite growth and transmission, we observed comparable, but more pronounced, effects upon the activation of male exflagellation and parasite transmission to those seen with *pyccr4-1*^*-*^ parasites (10-fold decrease in male activation on peak day and >4-fold reduction in transmission to mosquitoes, respectively) (Fig 1C and 1F). These exacerbated effects may be caused by the combined effects of a reduction in total parasite numbers due to the deletion of portions of PyCAF1 and a PyCCR4-1-dependent defect in male gametocyte development.

These effects upon asexual and sexual blood stage parasites could occur if this truncated form of PyCAF1 had reduced functionality due to an inability to associate with its complex. To assess this possibility, we raised specific antibodies to the N-terminus of the PyNOT1 scaffold protein and with them immunoprecipitated this complex, including PyCAF1ΔC::GFP (S3D Fig). This indicates that the CAF1 domain of PyCAF1 is sufficient for association with its complex and for its required functions. However, the remainder of the PyCAF1 protein contributes to the functions of PyCCR4-1, as this truncation phenocopies *pyccr4-1*^*-*^ and dPyCCR4-1 parasites.

**Fig 2:**
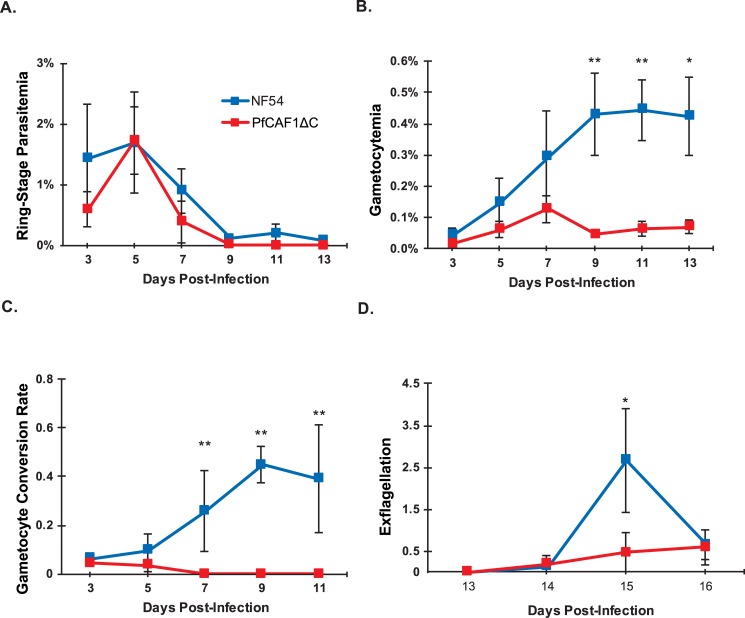
PfCAF1ΔC has decreased gametocyte conversion and exflagellation compared to wild type NF54 parasites. A) *P*. *falciparum* ring stage parasitemia and B) total gametocytemia counted in 10,000 RBCs were averaged over a minimum of two biological replicates. Ring stages were used to represent asexual parasitemia as they were most easily distinguishable from dead/dying asexual forms. C) Conversion rates were calculated as described previously [59] by taking the stage II-gametocytemia on Day T and dividing by ring stage parasitemia on Day T-2. D) Exflagellation events were counted under a 40x objective for 10 fields of view. Error bars represent standard error of the mean. Statistical differences between wild-type and PfCAF1ΔC parasites were assessed via a paired Wilcoxon test. * = p<0.05, ** = p<0.01.

To determine if the PfCAF1ΔC variant in human-infectious *P*. *falciparum* similarly impairs gametocytogenesis as was seen in rodent-infectious *P*. *yoelii*, the PfCAF1ΔC *piggyBac*-insertion parasite line was assessed for effects upon parasitemia, gametocytogenesis, as well as male gametocyte activation (Fig 2). The PfCAF1ΔC line exhibited significant decreases in gametocyte conversion, total gametocytemia, and exflagellation on the peak day as compared to wild-type *P*. *falciparum* NF54 strain parasites (Fig 2, S4 Table). These data support the observed *P*. *yoelii* phenotype and indicate that this conserved complex is important to sexual development across *Plasmodium* species.

### PyCCR4-1, PyCAF1, and PyNOT1 localize to discrete cytosolic granules

**Fig 3:**
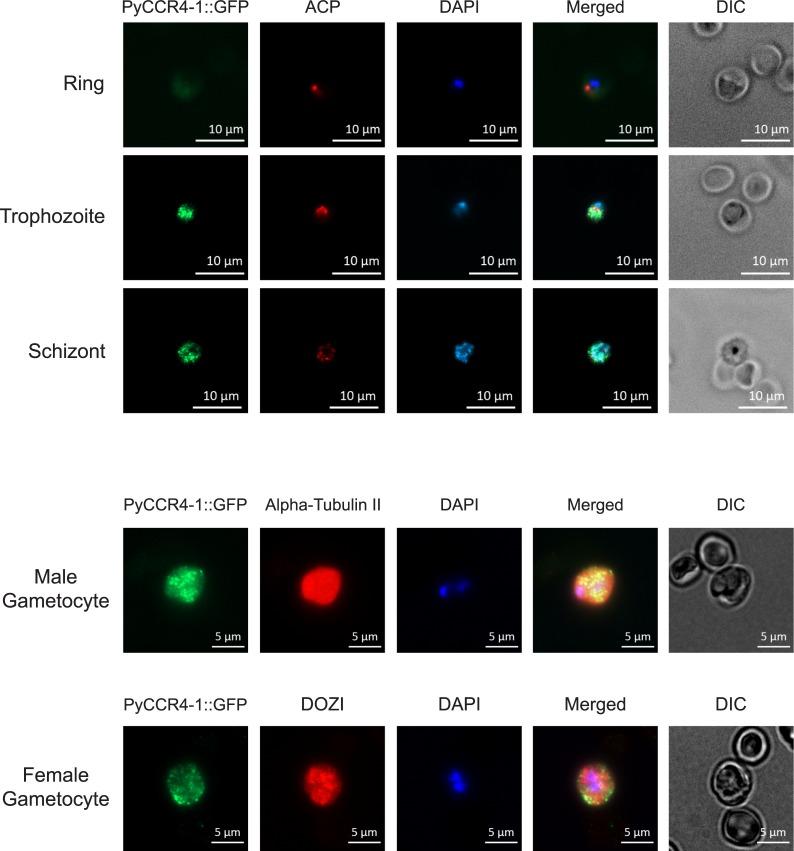
PyCCR4-1::GFP is expressed in cytosolic granules in asexual and sexual blood stage parasites. Representative images of parasites treated with DAPI and antibodies to GFP (to detect PyCCR4-1::GFP) or to stage-specific cellular markers (ACP, alpha-tubulin, or human DDX6 that cross-reacts with DOZI) are shown. Scale bars are 5 or 10 microns.

In eukaryotes, CCR4 and CAF1 function while in association with the other members of the CAF1/CCR4/NOT complex and is found in nuclear and cytosolic granular structures [20]. Because PyCCR4-1 lacks an obvious LRR domain by which it can associate with the rest of the complex, we used immunofluorescence and live fluorescence assays to further validate these interactions. First, using transgenic PyCCR4-1::GFP parasites, we observed that PyCCR4-1 localized to cytoplasmic puncta in asexual blood stage parasites, and is similarly localized in both male and female gametocytes (Fig 3). Moreover, this expression profile extends to oocysts, oocyst sporozoites, and salivary gland sporozoites, where PyCCR4-1 was seen both in cytosolic puncta and located diffusely throughout the parasite (S4A Fig). However, PyCCR4-1 was not detected above background in liver stage parasites (S4B Fig). Thus, the near constitutive expression and localization of PyCCR4-1 in cytoplasmic foci in *Plasmodium* resembles that of its orthologues in model eukaryotes. Next, using either full length PyCAF1::GFP or PyCAF1ΔC::GFP transgenic parasites, we observed a similar expression and localization pattern to that of PyCCR4-1::GFP (S4C and S4D Fig). Moreover, colocalization of PyCAF1ΔC::GFP and PyNOT1 signals were observed (S4E Fig). Together, these data further indicate that the truncated PyCAF1ΔC::GFP variant can remain associated with the rest of its complex and yet phenocopies these PyCCR4-1-associated effects.

### PyCCR4-1 affects important gametocyte and mosquito stage transcripts

**Fig 4:**
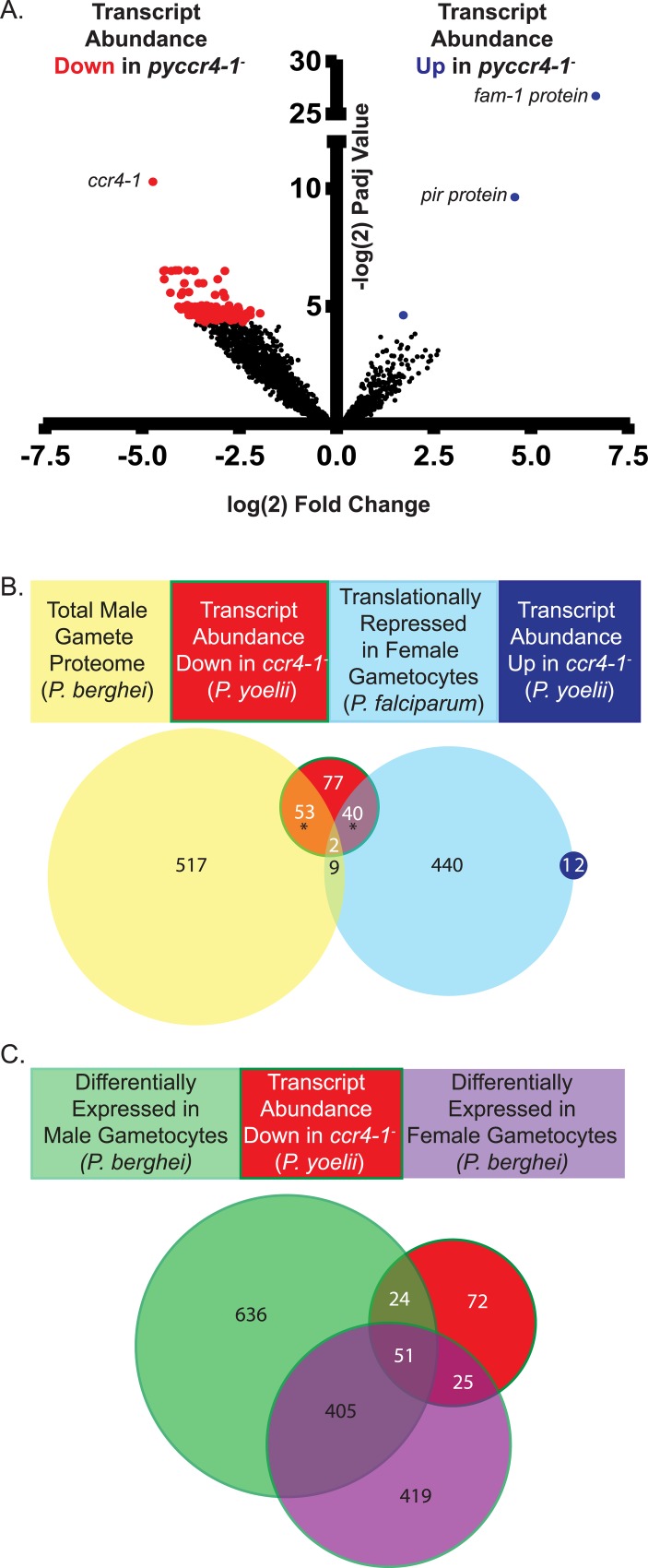
Transcripts with sex-specific or transmission-related functions are modulated by PyCCR4-1. A) A volcano plot showing statistically significant increases (blue), decreases (red), or no change (black) is provided. While few transcripts go up in abundance (from antigenically variant genes), nearly all affected transcripts decrease in abundance, thus indicating that PyCCR4-1 may play a role in preserving these mRNAs. B) In *pyccr4-1*^*-*^ gametocytes, 24% of differentially abundant transcripts are translationally repressed in female *P*. *falciparum* gametocytes and another one third of the transcripts are enriched in the male *P*. *berghei* gamete proteome [50, 69]. * = p<0.01 by Fisher test. C) In *pyccr4-1*^*-*^ gametocytes, transcripts that are differentially expressed in male and female *P*. *berghei* gametocytes are equally affected.

Because PyCCR4-1 is a putative deadenylase, we hypothesized that the defects in male gametocyte maturation and transmission observed in *pyccr4-1*^*-*^ and dPyCCR4-1 parasites may be attributed to PyCCR4-1 acting upon specific transcripts important to gametocytogenesis, gamete activation, and/or parasite transmission to mosquitoes. To determine the role of PyCCR4-1 in the regulation of transcripts in gametocytes, total comparative RNA sequencing (RNA-seq) was performed. Gametocytes from a wild-type line expressing GFP from the *p230p* dispensable locus (WT-GFP) and the *pyccr4-1*^*-*^ transgenic line were selected using sulfadiazine treatment, purified on an Accudenz gradient, and their RNA extracted for RNA-seq. Differential abundance of transcripts was assessed via DEseq2, and the p-adjusted value was used for all analyses (Fig 4A, S5 Table) [44, 45].

Nearly all (172 of 175) of the significantly affected transcripts (P-adjusted < 0.05, > 2 fold change) between WT-GFP and *pyccr4-1*^*-*^ parasites decreased in abundance in the *pyccr4-1*^*-*^ parasites, while only 3 transcripts increased in overall abundance (S6 Table). Many of these decreases in transcript abundance are for 55 mRNAs that encode male-enriched proteins, and thus these changes can likely be attributed to the production of fewer mature male gametocytes in the *pyccr4-1*^*-*^ transgenic line. However, the effect upon other transcripts, including those associated with female gametocytes, cannot be explained in this way (Fig 4B). Most notably, transcripts that encode for proteins involved in gamete function (*e*.*g*. GEST) and early mosquito stage development (*e*.*g*. p28, CITH, AP2-O, HMGB2, LAP2) decreased in abundance significantly in the absence of PyCCR4-1. While a catalog of translationally repressed transcripts is not available for *P*. *yoelii*, many of these transcripts are known to be translationally repressed in *P*. *falciparum* female gametocytes (Fig 4B). Interestingly, an ApiAP2 protein, recently identified as AP2-G3 (PY17X_1417400), that is important for gene expression in gametocytogenesis decreased in abundance 10-fold in *pyccr4-1*^*-*^ gametocytes [46]. Disruption of this ApiAP2 gene in *P*. *falciparum* by *piggyBac* transposon insertion resulted in the formation of no gametocytes and deletion of this gene in *P*. *yoelii* resulted in significantly reduced numbers of gametocytes [46, 47]. Effects upon this gene may have broad reaching effects on gametocyte development, and may contribute to the phenotypes observed here. Other transcripts-of-interest that decreased in abundance are those that encode for multiple uncharacterized RNA-binding proteins (PY17X_1203900, PY17X_1457300, PY17X_0923600), a second ApiAP2 protein (ApiAP2-O5, PY17X_1317000) and BDP2 (PY17X_1431000), an uncharacterized putative transcriptional activator (S6 Table) [47]. Additional analysis demonstrates that many of the transcripts that decrease in abundance in *pyccr4-1*^*-*^ gametocytes are differentially expressed in *P*. *berghei* gametocytes when compared to asexual parasites (Fig 4C) [48]. Together, we conclude that these differences in transcript abundance not only reflect a reduction in the number of mature male gametocytes, but also indicate that PyCCR4-1 is acting to preserve specific transcripts important for the gametocyte and early mosquito stage parasite.

### The CAF1/CCR4/NOT complex can specifically bind transcripts that are dysregulated in *pyccr4-1*^*-*^ parasites

**Fig 5:**
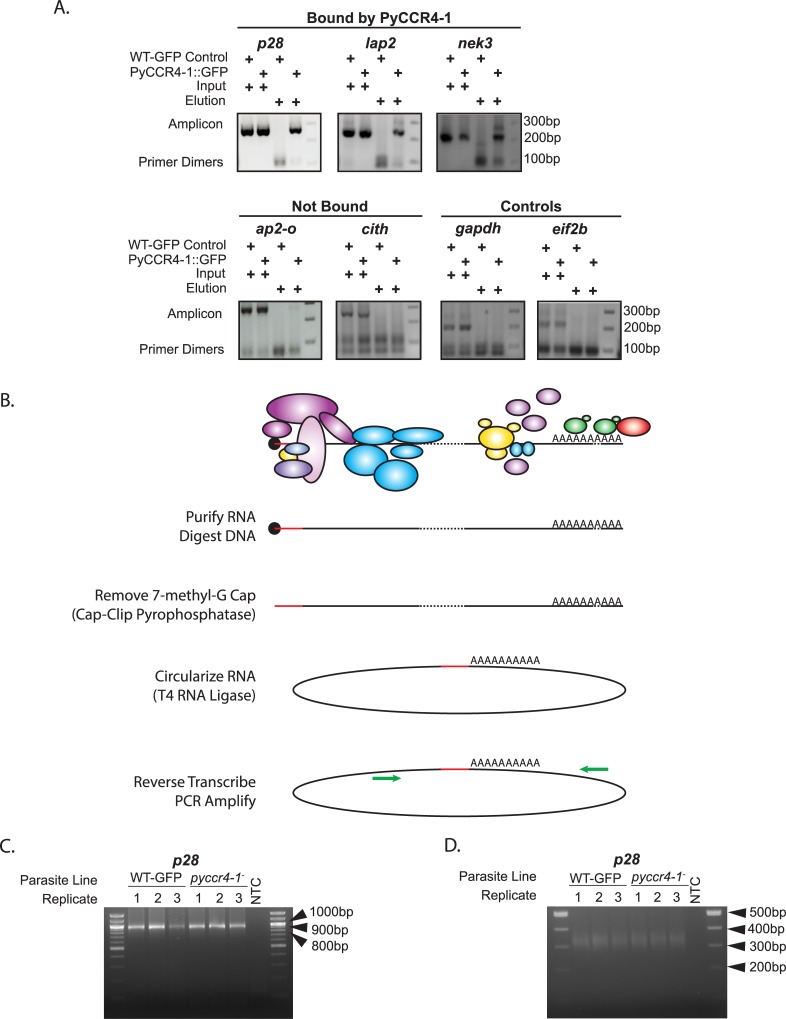
The CAF1/CCR4/NOT complex associates with some dysregulated transcripts but doesn’t grossly affect UTR/poly(A) tail length. A) Immunoprecipitation of PyCCR4-1::GFP allowed detection of the association of three selected transcripts that are affected by *pyccr4-1*^*-*^ (top), whereas other affected transcripts do not associate with PyCCR4-1::GFP (bottom, left). Two control transcripts that do not change upon deletion of *pyccr4-1* also do not interact with PyCCR4-1::GFP (bottom right). Shown are input and elution samples of a constitutive GFP-expressing clone and a PyCCR4-1::GFP clone. Amplicons and primer dimer bands are indicated with arrows. B) Circularized RT-PCR (cRT-PCR) allows for joining of the 5’ and 3’ ends of RNA following the removal of proteins and the 5’ 7-methylguanosine cap. C and D) Analysis of *p28* by cRT-PCR was conducted in both wild-type and *pyccr4-1*^*-*^ parasites to detect effects by PyCCR4-1 upon UTR and poly(A) tail length in purified gametocytes. Primers were designed within the coding sequences (B) or near the poly(A) tail (C) as defined by sequencing of cRT-PCR products from panel B, which allow observations of UTR and poly(A) tail lengths respectively. Three biological replicates and a No Template Control (NTC) are shown with NEB 100bp molecular weight ladder in parallel. Extended data is available in S5 and S6 Figs.

As transcript abundances could be affected directly by PyCCR4-1 and its complex, or indirectly through compensatory mechanisms such as gene buffering when the *pyccr4-1* gene is deleted [49], we investigated whether these dysregulated mRNAs were bound by the CAF1/CCR4/NOT complex. To this end, unfused GFP (expressed in WT-GFP parasites) and PyCCR4-1::GFP were immunoprecipitated from purified, transgenic gametocytes, and the association of co-precipitated transcripts was detected by RT-PCR. We found that PyCCR4-1::GFP interacted specifically with a number of selected transcripts that substantially change in abundance in *pyccr4-1*^*-*^ parasites, including *p28* (PY17X_0515900), *lap2* (PY17X_1304300), and *nek3* (PY17X_0603200) (Fig 5A, top row; S5A Fig). These transcripts are notable, as they are all important/essential for gametocytogenesis, transmission, or early mosquito stage development and include transcripts known to be expressed in male (*nek3*) and/or female (*p28*, *lap2*) gametocytes that are important for establishment of mosquito infections [48, 50–53]. However, not all dysregulated transcripts (*cith* and *ap2-o*) were found specifically associated with PyCCR4-1 (Fig 5A, bottom row; S5A Fig), suggesting that these effects likely result from a combination of both direct and indirect effects.

The direct effects that CCR4 can have on transcript abundance in other eukaryotes have resulted from deadenylation of a target transcript, or from translational repression by binding/tethering to the CAF1/CCR4/NOT complex [30]. To investigate whether the poly(A) tail length and/or UTRs were affected in the presence or absence of PyCCR4-1, circular-RT PCR (cRT PCR, illustrated in Fig 5B) was used to interrogate both a control transcript (*gapdh*, not affected by *ccr4-1* deletion, does not interact with CCR4-1), and an affected/bound transcript (*p28*) (Fig 5C and 5D, S5B and S5C Fig). In the absence of PyCCR4-1, there were no gross effects upon UTR/poly(A) tail lengths of these transcripts when visualized by PCR using primers that anneal near the start and stop codons (Fig 5B and 5C, oligonucleotides provided in S7 Table). Sequencing of cloned PCR products from both wild-type and *pyccr4-1*^*-*^ samples revealed the consistent composition of the 5’ and 3’UTRs, as well as the presence of a poly(A) tail (S6 Fig). Using the sequencing data, primers that anneal near to the poly(A) tail were used to assess the distribution of poly(A) tail lengths in the population. Using these primers, we did not observe any differences in poly(A) length between the wild-type and *pyccr4-1*^*-*^ populations, and are estimated to be ~75nt long (Fig 5D). These data indicate that the direct effect of PyCCR4-1 on these specific transcripts in gametocytes do not impact the poly(A) tail/UTR length, suggesting that the complex may be acting in other ways to preserve these transcripts.

## Discussion

**Fig 6:**
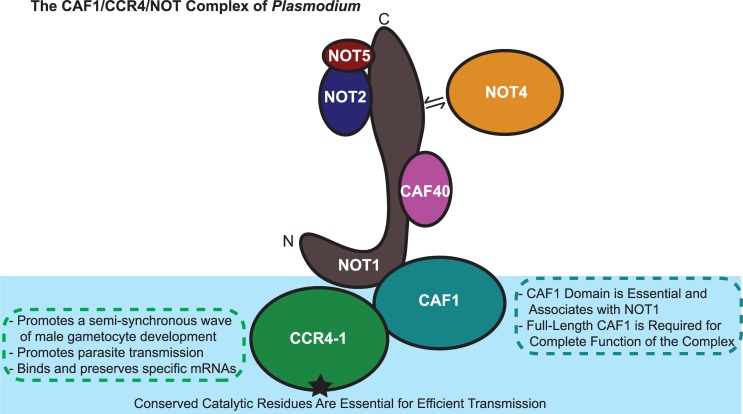
A model for the roles of PyCCR4-1 and PyCAF1 in gametocyte maturation and host-to-vector transmission is presented. The CAF1/CCR4/NOT complex in *Plasmodium* as identified through crosslinking IP-MS is shown. Contacts are inferred through previous studies in model organisms and sequence conservation of the interaction domains [20, 25, 70–72].

*Plasmodium* encodes few known specific transcription factors and a relatively over-represented number of RNA-binding proteins (10% of its predicted proteome) [11, 29]. One model suggests that *Plasmodium* has adapted these complementary regulatory mechanisms to achieve its preferred RNA homeostasis. Moreover, the malaria parasite also proactively transcribes a large number of genes before transmission, but does not produce the encoded proteins until transmission has occurred. This translational repressive mechanism has been shown to be imposed by members of the DOZI/CITH/ALBA complex, as well as by PUF2. Here, we demonstrate that the PyCCR4-1 and PyCAF1 members of the CAF1/CCR4/NOT complex play additional roles in either the preservation or expression of translationally repressed transcripts through direct and indirect means (Fig 6). Moreover, we find that PyCCR4-1 is also important for the development of the male gametocyte, as well as for the efficient transmission of gametocytes to the mosquito vector.

The composition and behaviors of the CAF1/CCR4/NOT complex are well-conserved in eukaryotes, but key species-specific differences exist. Our proteomic and bioinformatic analyses of *Plasmodium* species showed that many of the core components of this complex are shared between *Drosophila melanogaster*, *Saccharomyces cerevisiae*, *and Homo sapiens* (NOT1, NOT2, NOT5 (NOT3), NOT4, CCR4, CAF1 (POP2), and CAF40), but that some proteins are apparently not encoded at all (CNOT10) [29]. Additionally, while there are well-defined roles for this complex in both the nucleus and cytoplasm of other eukaryotes, we find by IFA that the vast majority of PyCCR4-1, PyCAF1, PyNOT1, and by inference the entire complex, localizes to discrete cytosolic granules throughout most of the *Plasmodium* life cycle. For this reason, we have focused our analyses here upon the known cytoplasmic roles of the complex, but additional work to define possible nuclear functions of the CAF1/CCR4/NOT complex is certainly warranted.

Previous studies of CCR4 in other eukaryotes have demonstrated that CCR4 can act as a translational repressor, and that its catalytic residues and deadenylase activity are implicated in its repressive functions [30, 54, 55]. Our results demonstrate that PyCCR4-1 helps to preserve translationally repressed transcripts and are suggestive of similar roles in *Plasmodium* (Fig 4A). Furthermore, transgenic parasites that lack *pyccr4-1*, that express a putatively dead catalytic variant of PyCCR4-1, or that express a truncated variant of PyCAF1 (PyCAF1ΔC) demonstrate the same phenotype, and thus we conclude that the catalytic residues of the PyCCR4-1 protein and the portion of CAF1 C-terminal to the CAF1 domain are required for this regulatory function (Figs 1 and 6). As the maturation of male gametocytes is substantially promoted by the presence of catalytically active PyCCR4-1, it is unsurprising that one third of the transcripts that PyCCR4-1 affects encode proteins that are present in a *P*. *berghei* male gamete proteome (Fig 4). It should be noted that the PyCCR4-1 catalytic mutant (dCCR4-1) has a slightly stronger effect upon gametocytogenesis, gametogenesis, and parasite transmission than does the deletion of *pyccr4-1*. This effect could result if dCCR4-1 is still able to bind transcripts and prevent other proteins from assembling onto them, thus acting as a dominant negative variant.

Expression of a truncated CAF1 variant in *P*. *yoelii* or *P*. *falciparum* produced similar phenotypes during gametocytogenesis and male gametocyte activation (Figs 1 and 2). Immunoprecipitation and IFA analyses of PyNOT1 and PyCAF1ΔC showed that this could not be explained by the disruption of their interaction. This work also defines the CAF1 domain as the essential portion of the PfCAF1 and PyCAF1 proteins, and that sequences C-terminal of it play additional functional, but non-essential roles.

During sexual development, we find that PyCCR4-1 binds to multiple transcripts essential for gametocyte development and host-to-vector transmission, which are dysregulated in *pyccr4-1*^*-*^, parasites. We hypothesize that PyCCR4-1 is interacting with and acting upon these transcripts to preserve them for use post-transmission. Circular RT-PCR demonstrated that UTR and poly(A) lengths are not affected in *pyccr4-1*^*-*^ transgenic parasites for a dysregulated, female transcript (*p28*). Global analyses of poly(A) tail length have been demonstrated in other eukaryotes, and could provide evidence for whether this phenomenon holds for all dysregulated transcripts. Therefore, we posit that PyCCR4-1 and its complex can be utilizing functions independent of deadenylation for these regulatory functions. For instance, tethering of transcripts to the CAF1/CCR4/NOT complex can induce repression of a target transcript in other systems, even in the absence of the deadenylation of those transcripts [30]. Taken together, these data indicate that the *Plasmodium* CAF1/CCR4/NOT complex provides key functions in the regulation of specific transcripts to promote coordinated male gametocyte maturation and parasite transmission.

## Materials and methods

Extended versions of materials and methods are provided in S1 File.

### Ethics statement

All animal care strictly followed the Association for Assessment and Accreditation of Laboratory Animal Care (AAALAC) guidelines and was approved by the Pennsylvania State University Institutional Animal Care and Use Committee (IACUC# 42678–01). All procedures involving vertebrate animals were conducted in strict accordance with the recommendations in the Guide for Care and Use of Laboratory Animals of the National Institutes of Health with approved Office for Laboratory Animal Welfare (OLAW) assurance.

### Experimental animals

Six-to-eight week old female Swiss Webster mice from Harlan (recently acquired by Envigo) were used for all of the experiments in this work. *Anopheles stephensi* mosquitoes (obtained from the Center for Infectious Disease Research; Seattle, WA) were reared at 24C and 70% humidity and were used to cycle *Plasmodium yoelii* (17XNL strain) parasites.

### Production of transgenic parasite lines

Transgenic *Plasmodium yoelii* (17XNL strain) parasites were created using targeting sequences to incorporate sequence into the target gene using double homologous recombination using standard procedures [56]. Parasite genomic DNA was purified (QIAamp DNA Blood Kit, Qiagen, Cat# 51106) and genotyping PCR was performed to assess the ratio of WT to transgenic parasites present. Clonal parasite populations were produced using limiting dilution cloning. The PfCAF1ΔC line was previously generated as described [26]. Validation of CAF1 transcript expression was performed via RT-PCR on 100ng DNase-treated RNA from PfCAF1ΔC and NF54-control parasites using primer sets supplied in S7 Table.

### Production and accudenz purification of *P*. *yoelii* schizonts and gametocytes

To produce schizonts in culture, infected Swiss Webster mice were exsanguinated by cardiac puncture and the blood was collected into complete RPMI (cRPMI), spun at 200 *xg* for 8 min to remove the serum, and then cultured in 30 ml cRPMI in a 5% CO_2_, 10% O, 85% N gas mixture for 12 hours at 37C. Cultures were underlayed with 10 ml of 17% w/v Accudenz in 5 mM Tris-HCl (pH 7.5@RT), 3 mM KCl, 0.3mM disodium EDTA, 0.4x PBS (without calcium and magnesium) and spun at 200 *xg* for 20 min with no brake [56]. Parasites were collected from the interface between the Accudenz and cRPMI layers, transferred to a fresh conical tube, supplemented with an equal volume of additional cRPMI, and spun for 10 min at 200 *xg*. The supernatant was then removed and the parasite pellet processed for downstream applications.

Gametocytes were produced by treatment of the mice at 1% parasitemia with 10 mg/L sulfadiazine (VWR, Cat# AAA12370-30) in their drinking water for two days prior to exsanguination. The blood was maintained in warm (37C) cRPMI to prevent activation of gametocytes and parasites were purified as described above.

### *P*. *falciparum* gametocyte production

Gametocyte-producing cultures were established as described previously [57] with some modification. Briefly, starter cultures of wild-type *P*. *falciparum* NF54 and PfCAF1ΔC were grown to ~5% parasitemia in standard culture conditions in cRPMI supplemented with 25 mM HEPES, 0.2% D-glucose, 200 uM hypoxanthine, 0.2% w/v sodium bicarbonate, and 10% v/v heat-inactivated human serum at 6% hematocrit in a tri-gas incubator (5% CO_2_, 5% O_2_) at 37C. On Day 0, starter cultures were then used to inoculate 75 cm^2^ flasks (15ml culture volume at 6% hematocrit) in technical duplicate for each line at 0.5% parasitemia. Parasites were cultured for 17 days with daily media changes and no fresh addition of blood. Samples were taken to monitor parasite development starting at Day 3 post-infection and then every 48 hours until Day 13 post-infection, determined through Giemsa-stained smears. At seven days post-infection, technical replicate flasks were combined into one flask, which was maintained for the duration of the experiment. Samples were assessed in biological triplicate.

### PyDD recombinant protein expression and purification

Coding sequence for a domain of PyDD (PY17X_0418900, “PyDDD” = AA1845-2334) was generated by IDT as a codon-optimized gene block (gBlock) for expression from a modified pET28b+ vector (pSL0220) in *E*. *coli* BL21 (DE3) pLysS CodonPlus bacteria. Protein was purified first by standard Ni-NTA and then glutathione resin approaches (details provided in S1 File). Purity was confirmed to be >90% by SDS-PAGE by Coomassie Blue staining. Antibodies were generated in rabbits (screened for pre-immune sera with minimal background reactivity) by Pocono Rabbit Farm and Laboratory (Canadensis, PA).

### Live fluorescence and IFA microscopy

PyCCR4-1 and PyCAF1 expression in blood stages, oocyst sporozoites, salivary gland sporozoites and liver stages was observed by an indirect immunofluorescence assay (IFA), and expression in day seven oocysts was observed by live fluorescence. All samples for IFA were prepared as previously described, with all details provided in S1 File [58]. Fluorescence and DIC images were taken using a Zeiss fluorescence/phase contrast microscope (Zeiss Axioscope A1 with 8-bit AxioCam ICc1 camera) using a 40X, 63X, or 100X oil objective and processed by Zen imaging software.

### Measurement of blood stage growth kinetics

Cryopreserved blood infected with either wild-type (Py17XNL), *pyccr4-1*^*-*^, dCCR4-1, or PyCAF1ΔC parasites were injected intraperitoneally into Swiss Webster starter mice and parasitemia was allowed to increase to 1%. This blood was extracted via cardiac puncture and diluted in RPMI to 10,000 parasites per 100 ul (CCR4-1, CAF1ΔC) or 1,000 parasites per 100ul microliter (dCCR4-1). One hundred microliters was injected intravenously (IV) into three mice per replicate for each parasite line. Three biological replicates were conducted, each with three technical replicates. Parasitemia was measured daily by giemsa-stained thin blood smears. Centers of movement/exflagellation centers were also measured daily via wet mount of the blood incubated at room temperature for 10 min by counting the number of exflagellating male gametocytes in a confluent monolayer per 400x field (40x objective x 10x eyepiece).

*P*. *falciparum* ring stage parasitemia and total gametocytemia were calculated every two days starting on Day 3 post-infection by averaging counts in 10,000 RBCs across a minimum of two biological replicates (provided in S6 Table). Sexual conversion was calculated as described previously [59] by taking the stage II-gametocytemia on Day T and dividing by ring stage parasitemia on Day T-2. Samples were taken for exflagellation assays on days 13, 14, 15, and 16 post-infection. Two-hundred microliter samples were taken from each flask and spun down at 300 *xg* for 30 seconds. Supernatant was removed and a 20 ul aliquot of remaining blood pellet was mixed with 20 ul of heat-inactivated human serum previously warmed to 37°C. The mixture was then allowed to incubate at room temperature for 15 min, after which exflagellation events were counted under 40x magnification for 10 fields-of-view.

### Flow cytometry gametocyte counts

Cryopreserved blood infected with either wild- type (Py17XNL), *ccr4-1*^*-*^, dCCR4-1, or CAF1ΔC parasites was injected intraperitoneally into starter mice and transferred as above (10,000 parasites/100ul). On Day 5, gametocytes were produced by treatment of the mice with 10 mg/L sulfadiazine (VWR, Cat# AAA12370-30) in their drinking water for two days. Blood was collected by cardiac puncture and maintained in warm cRPMI to prevent activation of gametocytes and spun at 37°C. Blood was then fixed, passed through a cellulose column and stained as described above for IFA. Parasites were stained with the following primary antibodies: mouse anti-PvBIP Clone 7C6B4 (1:1000; [60]) and rabbit anti-PyDynein Heavy Chain Delta Domain (“PyDDD”, PY17X_0418900 AA: 1845 to 2335)) (1:1000, Pocono Rabbit Farm & Laboratory, Custom PAb), along with goat anti-mouse conjugated to AF594 (Fisher Scientific, A11012) and goat anti-rabbit conjugated to AF647 (Fisher Scientific, PIA32733) secondary antibodies. These were then analyzed on a LSR Fortessa (BD) in tube mode and collected samples were analyzed in FlowJo.

### Mosquito transmission studies

Cryopreserved blood infected with either wild-type (Py17XNL), *ccr4-1*^*-*^, dCCR4-1, or CAF1ΔC parasites was injected intraperitoneally into starter mice and transferred as above. Centers of movement were checked daily as above and mice were fed to mosquitoes on the peak day of exflagellation (day 5). Mosquito midguts were dissected at D7 post feed and analyzed for the prevalence of infection and oocyst numbers by microscopy. Mosquito midguts (day 10) or salivary glands (day 14) were dissected, ground, and sporozoite numbers counted.

### Immunoprecipitations, western blotting, and mass spectrometric proteomics

Parasite pellets (schizonts) were crosslinked in 1% v/v formaldehyde and lysed using RIPA lysis buffer with a 1x protease inhibitor cocktail and 0.5% v/v SUPERase In, dounce homogenization with a tight pestle, and sonication. The parasite lysate was then precleared using streptavidin-coated dynabeads was immunoprecipitated using a biotin-conjugated antibody (anti-GFP or anti-PyNOT1) loaded on streptavidin-coated dynabeads for three hours at 4C with rotation. The beads were washed with modified RIPA wash buffer (50 mM Tris-HCl (pH 8.0@RT), 1 mM EDTA, 150 mM NaCl, 1% v/v NP40) once and then transferred to a new tube. The beads were washed 3 more times with modified RIPA wash buffer and then eluted at 45C overnight in a heat block. Samples were quality controlled by western blotting, and then subjected to tryptic digest and LC/MS/MS identification (Harvard Proteomics Core, run parameters listed in S1 File). The data was processed using the Trans-Proteomic Pipeline (TPP) [61] as described previously with few modifications [19]. Spectra were searched against reference sequences downloaded in February 2016 from *Plasmodium yoelii* 17X (PlasmoDB, v27), mouse (Uniprot), and common contaminants (Common repository of adventitious protein sequences, [62] and randomized decoys generated through TPP. X!Tandem and Comet searches were combined in iProphet [63] and protein identifications were determined by Peptide Prophet. Only proteins with a highly stringent false positive error rate of less than 1% are reported. To combine replicate proteomics datasets, SAINT version 2.5.0 was used [64]. Only proteins with SAINT scores below 0.1 (most stringent) or 0.35 (stringent) were considered significant hits and included in the analyses, as used previously [2, 4, 6]. The total proteome of Py17XNL mixed blood stages was determined using the same workflow (Penn State Proteomics Core).

### Total and comparative RNA-seq

Gametocytes were produced, collected, and purified by an Accudenz gradient, as above. Infected RBCs were lysed with saponin, washed with 1xPBS, and released parasites were then lysed immediately using the QIAgen RNeasy Kit using the manufacturer’s protocol with the additional on-column DNaseI digestion. RNA yields were quantified spectrophotometrically by NanoDrop, and RNA samples were further quality controlled (BioAnalyzer) and used to create barcoded libraries (Illumina TruSeq Stranded mRNA Library). An equimolar pool of all samples was made and 100 nt single end read sequencing was performed on an Illumina HiSeq 2500 in Rapid Run mode. The resulting data was mapped to the *P*. *yoelii* 17XNL strain reference genome (plasmodb.org, v32 using Tophat2 in a local Galaxy instance (version .9). Gene and transcript expression profiles for both WT-GFP and *ccr4-1*^*-*^ assemblies were generated using htseq-count (Galaxy version 0.6.1galaxy3) [65] using the union mode for read overlaps. Count files were merged and compared using DESeq2 (Galaxy version 2.11.39 [66]). Six biological replicates were used for the WT transcriptomic profile, while four replicates were used in for the *ccr4-1*^*-*^ profiles. These were analyzed by a mean fit type with outlier replacement turned on to normalize the variance between the count files. The P-adjusted value was used for all analyses.

### Circular reverse transcription PCR (cRT-PCR)

RNA was isolated from purified *P*. *yoelii* wild type or *pyccr4-1*^*-*^ gametocytes by TRIzol/chloroform extraction and extensive DNaseI digestion. The 7-methylguanosine cap was removed from 10ug of total RNA using 2.5U Cap-Clip Acid Pyrophoshatase in 1xCap-Clip Buffer supplemented with 10U Murine RNase Inhibitor at 37C for 1 hour. Treated RNA was TRIzol extracted, precipitated, dried, and then circularized with T4 RNA Ligase in T4 DNA Ligase buffer supplemented with 10% w/v PEG8000 and 10U Murine RNase Inhibitor at 16C for 24 hours. RNA was purified, precipitated, dried, and then subjected to reverse transcription using SuperScript IV and gene-specific primers (S7 Table). Specific PCR amplification of *gapdh* and *p28* sequences from the resulting cDNA was conducted using Phusion polymerase (NEB) and gene specific primers (S7 Table).

### Statistical analyses

Statistical differences between *P*. *yoelii* wild-type and transgenic parasites were assessed via a two-tailed t-test on Graphpad Prism. Statistical differences between *P*. *falciparum* wild-type and PfCAF1ΔC parasites were assessed via a paired Wilcoxon test using R v. 3.3.1 [67] with p < 0.05 indicating statistical significance.

### Data availability statement

All data is publically available on common data repositories. Proteomics data is accessible at the ProteomeXchange Consortium via the PRIDE partner repository with the dataset identifier PXD007042 [68]. Transcriptomics data (both RAW and processed files) is accessible at the GEO repository (Accession #GSE101484). Details of datasets and identifiers are available in S1 File.

## Supporting information

S1 Fig Bioinformatic and reverse genetic characterization of CCR4-family proteins. A) Schematics of the four bioinformatically predictable CCR4 domain-containing proteins (CCR4-1, 2, 3, and 4) of *Plasmodium* species are shown. The four proteins with identified exonuclease-endonuclease-phosphatase domains (shaded white rectangles) are shown to scale with their domain architecture, introns (gaps) and exons (rectangles). Also shown are E-values for their EEP domain based upon their alignment with CCR4 (PLN03144) via the Conserved Protein Domain Database. B) The four bioinformatically predictable CCR4 domain-contains proteins from *P*. *yoelii*, the catalytically dead PyCCR4-1 (dPyCCR4-1), CCR4-1 from *P*. *falciparum*, and orthologues from *S*. *cerevisiae*, human, and mouse were aligned using EMBL Clustal Omega. Shown is the region around the catalytic residues of CCR4. Amino acids noted in red font are the two catalytic residues, while those noted in white font with black highlighting are the two residues that were changed to create dPyCCR4-1. C-F) Genotyping PCR of (C) *pyccr4-1*^*-*^, (D) *pyccr4-2*^*-*^, (E) *pyccr4-3*^*-*^, and (F) *pyccr4-4*^*-*^ transgenic parasites. Successful genetic deletions were created using double homologous recombination of the targeting sequence consisting of ~750bp on either side of the ORF. Genotyping was performed by PCR on parasites cloned by limiting dilution using the primers indicated (listed in S7 Table). Independent clones were compared to Py17XNL wild-type control genomic DNA, a no template control, and a plasmid positive control in parallel.
(PDF)

S2 Fig Production and phenotyping of transgenic parasite lines. A) Asexual blood stage growth was monitored for two *pyccr4-1*^*-*^ transgenic clonal lines compared to a WT-GFP control line over the entire course of an infection. No significant difference in growth kinetics was observed. B) Gametocyte counts were performed using flow cytometry. Asexual stage parasites were removed with two days of sulfadiazine treatment and WBC’s were removed using a cellulose column. PyDDD high and BIP + cells were scored as mature male gametocytes and DDD mid and BIP+ cells were scored as immature or female gametocytes. No red blood cells were excluded in this analysis, and thus permitted measurement of gametocytemia. A PyDDD promoter driving GFP was used to establish gating of mature male gametocytes. PyDDD+ cells were FACS selected and observed to be male gametocytes by Giemsa staining and could undergo gametogenesis (exflagellation assay). C) Mature male or immature/female gametocytemia were counted by flow cytometry for wild-type and transgenic parasite lines in this study. D) Genotyping PCR of *pyccr4-1*::*gfp* transgenic parasites was performed by PCR on parasites as described in S1 Fig. Expression of PyCCR4-1::GFP was detected at ~250kDa by western blotting of immunoprecipitated material. E) Genotyping PCR of dPyCCR4-1 transgenic parasites is shown. A successful replacement of the PyCCR4-1 catalytic residues were created using double homologous recombination to insert a C-terminal GFP tag and stop codon following the PyCCR4-1 stop codon. Genotyping was performed by PCR on parasites as described in S1 Fig. Sequencing results are shown demonstrating the appropriate base change to substitute alanine for these two amino acids has occurred. F) Mosquitoes fed upon mice infected with *pyccr4-1*^*-*^ parasites performed 2 days after the peak day of exflagellation (D7). The number of oocysts per infected mosquito on day seven post-infectious blood meal are plotted. Data represents at least 20 dissected mosquitoes per biological replicate conducted in triplicate. Error bars represent the standard error of the mean.
(PDF)

S3 Fig A) Genotyping PCR of *pycaf1*^*-*^ transgenic parasites. An attempt at the deletion of *pycaf1* by double homologous recombination using targeting sequences consisting of ~750bp on either side of the ORF is depicted. Genotyping was performed by PCR as described in S1 Fig. B) A *P*. *falciparum* line carrying a *piggyBac* transposon inserted after the CAF1 deadenylase domain makes a truncated transcript. A schematic of RT-PCR primers aligned to the CAF1 ORF is provided as a reference, with the site of the *piggyBac* disruption indicated by a dotted line. C) Genotyping PCR of a *pycaf1* disruptant transgenic parasites is shown. A successful disruption of *pycaf1* was created using double homologous recombination to insert a C-terminal GFP tag and stop codon following the CAF1 domain (PyCAF1ΔC). Genotyping was performed by PCR as described in S1 Fig. D) Immunoprecipitations were performed on three different parasite backgrounds, PyWT-GFP, PyCAF1::GFP, and PyCAF1ΔC using either an anti-GFP or anti-NOT1-G antibody. These were then probed with a different anti-GFP antibody than the one used for immunoprecipitation. A 2 min exposure and 10 minute exposure are provided to allow visualization of GFPmut2, full length PyCAF1::GFP, and PyCAF1ΔC.
(PDF)

S4 Fig Expression and localization of PyCCR4-1, PyCAF1, PyCAF1ΔC, and PyNOT1 by immunofluorescence. A, B) PyCCR4-1::GFP is expressed in mosquito stage parasites but is not detectable in liver stage parasites. Representative images are shown of A) oocyst sporozoites, salivary gland sporozoites, and B) 24 hour and 48 hour liver stage parasites treated with DAPI and antibodies to GFP (to detect PyCCR4-1::GFP) or to stage-specific cellular markers (CSP, ACP, alpha-tubulin, or DOZI). Oocysts were imaged by live fluorescence. Scale bars are either 20 microns (oocysts), 5 microns (sporozoites), or 10 microns (liver stage parasites). C, D, E) PyCAF1::GFP and PyCAF1ΔC::GFP parasites were imaged by IFA as described in Fig 3 using anti-GFP, anti-ACP, and anti-PyNOT1 antibodies.
(PDF)

S5 Fig Extended data related to Fig 5. A) Control reactions of samples not treated with reverse transcriptase (-RT) are provided in addition to the +RT experimental samples for all assays. Assessment of *gapdh* (B) and *p28* (B,C) by cRT-PCR is also provided as a control.
(PDF)

S6 Fig Sanger sequencing of cRT-PCR products from the circularized p28 transcript using primers that anneal within the coding sequence (upper case) permitted identification of the 5’ (red font, lower case) and 3’ UTRs (blue font, lower case), as well as the poly(A) tail (black font, lower case, underlined). Sequencing could not extend robustly through the poly(A) tail to provide an exact length from either forward or reverse sequencing primers (denoted by dashes). Sequences of the circularized gene product are provided from a cloned PCR product that is representative of UTRs from both wild-type and *pyccr4-1*^*-*^ samples.
(PDF)

S1 File An extended version of the materials and methods used for this study is provided here. (DOCX)

S1 Table Measurements of transmission-related phenotypes for deletions of *pyccr4-1*^*-*^, *pyccr4-2*^*-*^, *pyccr4-3*^*-*^, *pyccr4-4*^*-*^, dPyCCR4, and PyCAF1ΔC. Average values for each clone are shown for each replicate. Centers of movement/exflagellation centers are shown as an average of the number of exflagellating males per field in ten 400x fields. Prevalence of mosquito infections, oocyst counts, and sporozoite counts are all averages from at least 20 mosquitoes per replicate conducted in biological triplicate. Additional information on methods and data listed is provided in a README tab. Male and female gametocyte counts are shown for wild-type parasites expressing GFPmut2 from the safe harbor *p230p* locus and *pyccr4-1*^*-*^ transgenic parasite lines.
(XLSX)

S2 Table The total proteomes of PY17XNL wild-type parasites and *pyccr4-1*^*-*^ parasites are provided as their RAW output, FDR 1% cutoff lists, and lists of the *Plasmodium* proteins detected within the 1% FDR cutoff for each parasite type. (XLSX)

S3 Table Output files from TPP (The Trans-Proteomic Pipeline) are shown in individual tables for each control and experimental replicate. The output from the SAINT IP-MS data analysis is also shown with the PyCCR4-1::GFP bait protein manually added. Identified proteins are sorted by SAINT Scores, with highly stringent (< 0.1) being unshaded and stringent (0.1 to 0.35) hits shaded light grey, and all proteins with SAINT scores >0.35 shaded dark gray. The output files from TPP (The Trans-Proteomic Pipeline) are shown in individual tables for each control and experimental replicate. The output from the SAINT IP-MS data analysis is also shown with the PyCAF1ΔC::GFP bait protein manually added. Identified proteins are sorted by SAINT Scores, with highly stringent (< 0.1) being unshaded and stringent (0.1 to 0.35) hits shaded light grey, and all proteins with SAINT scores >0.35 shaded dark gray.
(XLSX)

S4 Table Measurements of *P*. *falciparum* transmission-related phenotypes. Data are shown for each replicate. Additional information is provided in a README tab.
(XLSX)

S5 Table The raw and annotated outputs from DEseq2 comparison of transcript abundance from four biological replicates of *pyccr4-1*^*-*^ parasites vs six biological replicates of Py17XNL wild-type parasites is provided. Additional information on data listed is provided in a README tab.
(XLSX)

S6 Table Genes associated with translationally repressed transcripts in female gametocytes (31), male gamete-enriched proteins (32), and transcripts that are affected by deletion of *pyccr4-1* are listed. Comparisons of these lists were used to generate Venn diagrams (Fig 4). The Input tab is the gene ID’s from each input and the Output tab contains the independent and overlapping fields of the Venn diagram in Fig 4.
(XLSX)

S7 Table Oligonucleotides that were used to generate and genotype transgenic parasites are shown. Upper case letters indicate nucleotides that are homologous to the native genomic sequence, while lower case letters are not.
(XLSX)
