# Supplementary figures and images for: Plasmodium male gametocyte development and transmission are critically regulated by the two putative deadenylases of the CAF1/CCR4/NOT complex
Source: PLoS Pathog. 2019 Jan 31;15(1):e1007164. doi: 10.1371/journal.ppat.1007164 (PMC6355032; doi:10.1371/journal.ppat.1007164)

S1 Figure: Hart *et al.*  
A.

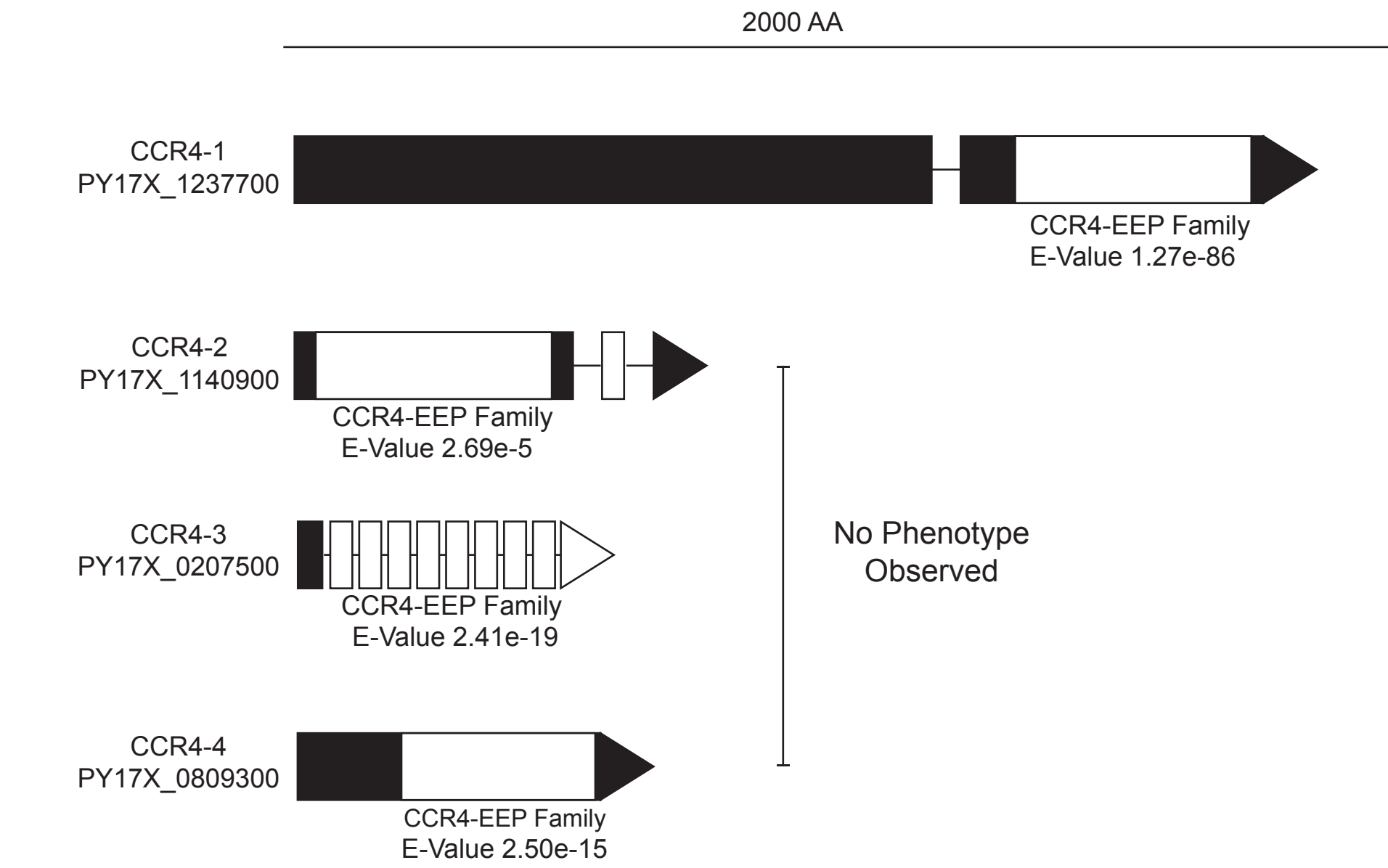

B.

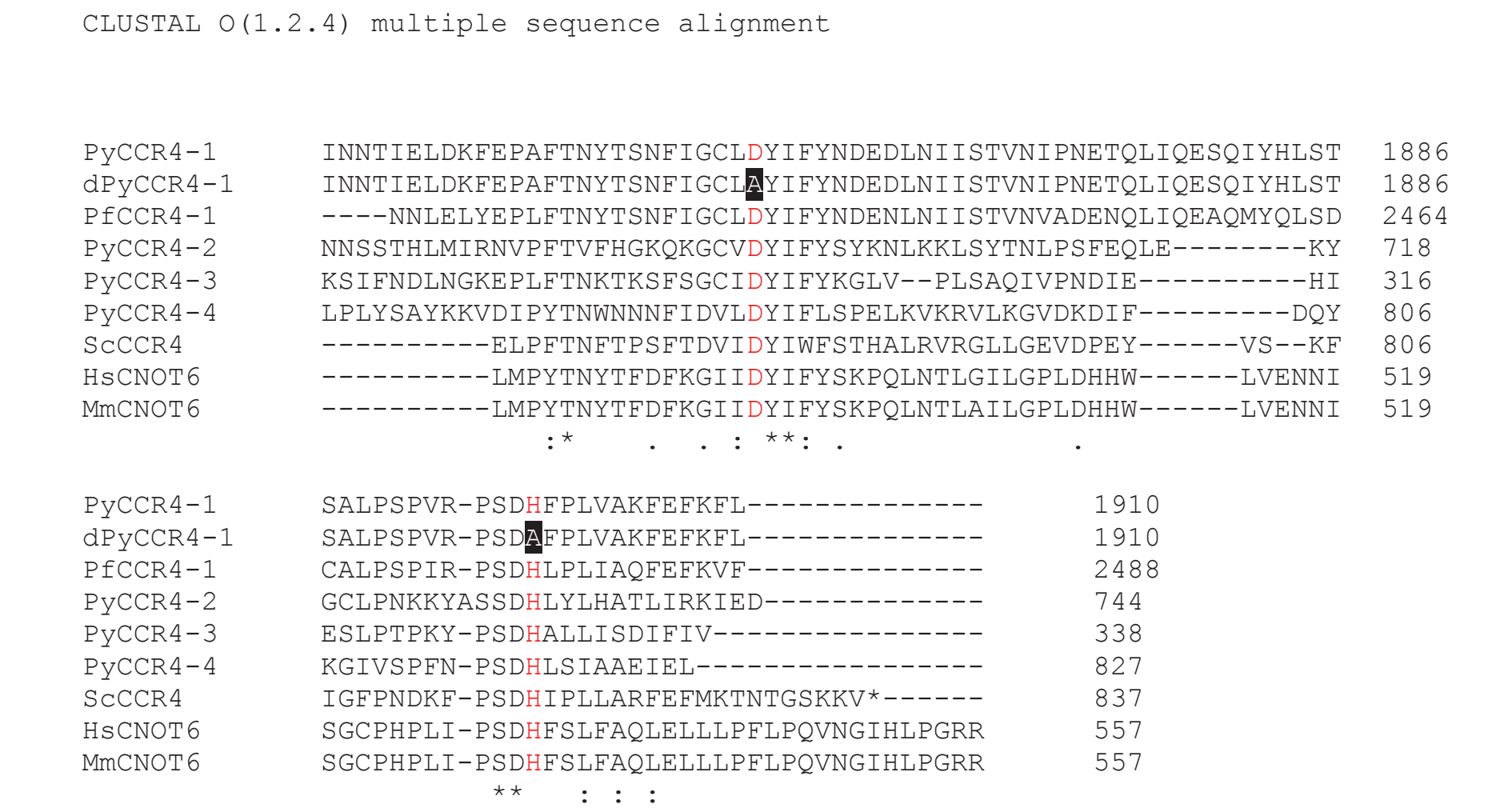

C.

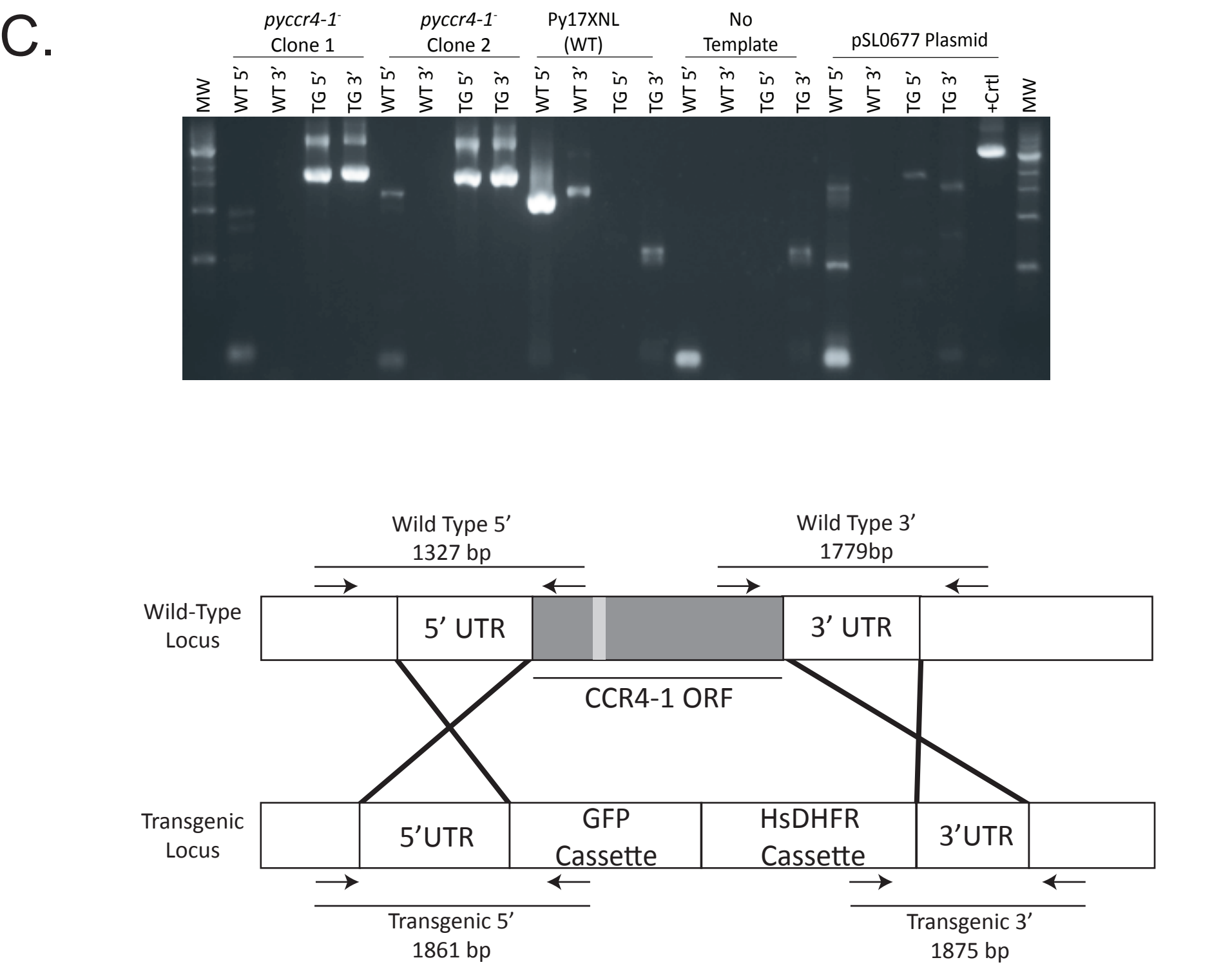

D.

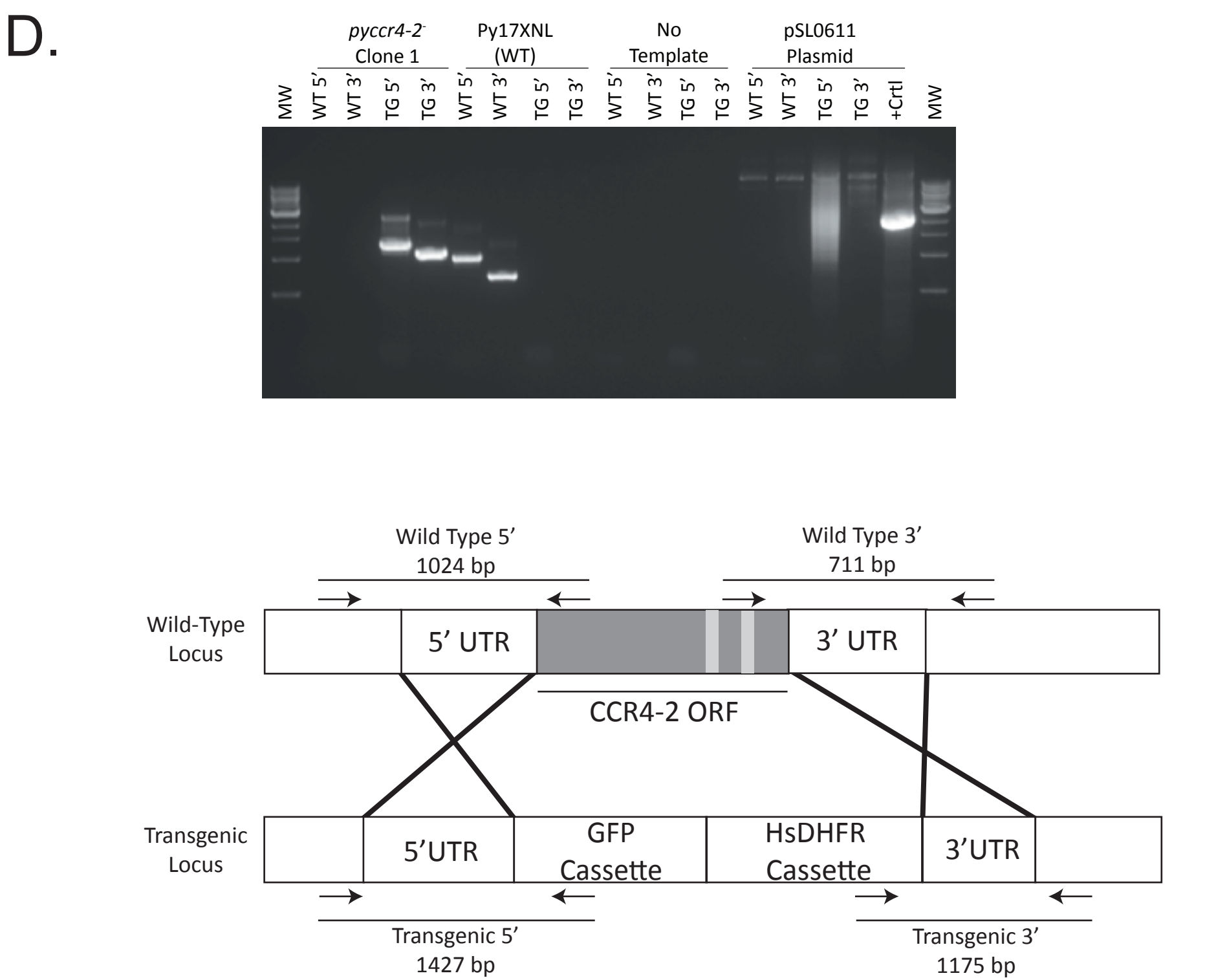

E.

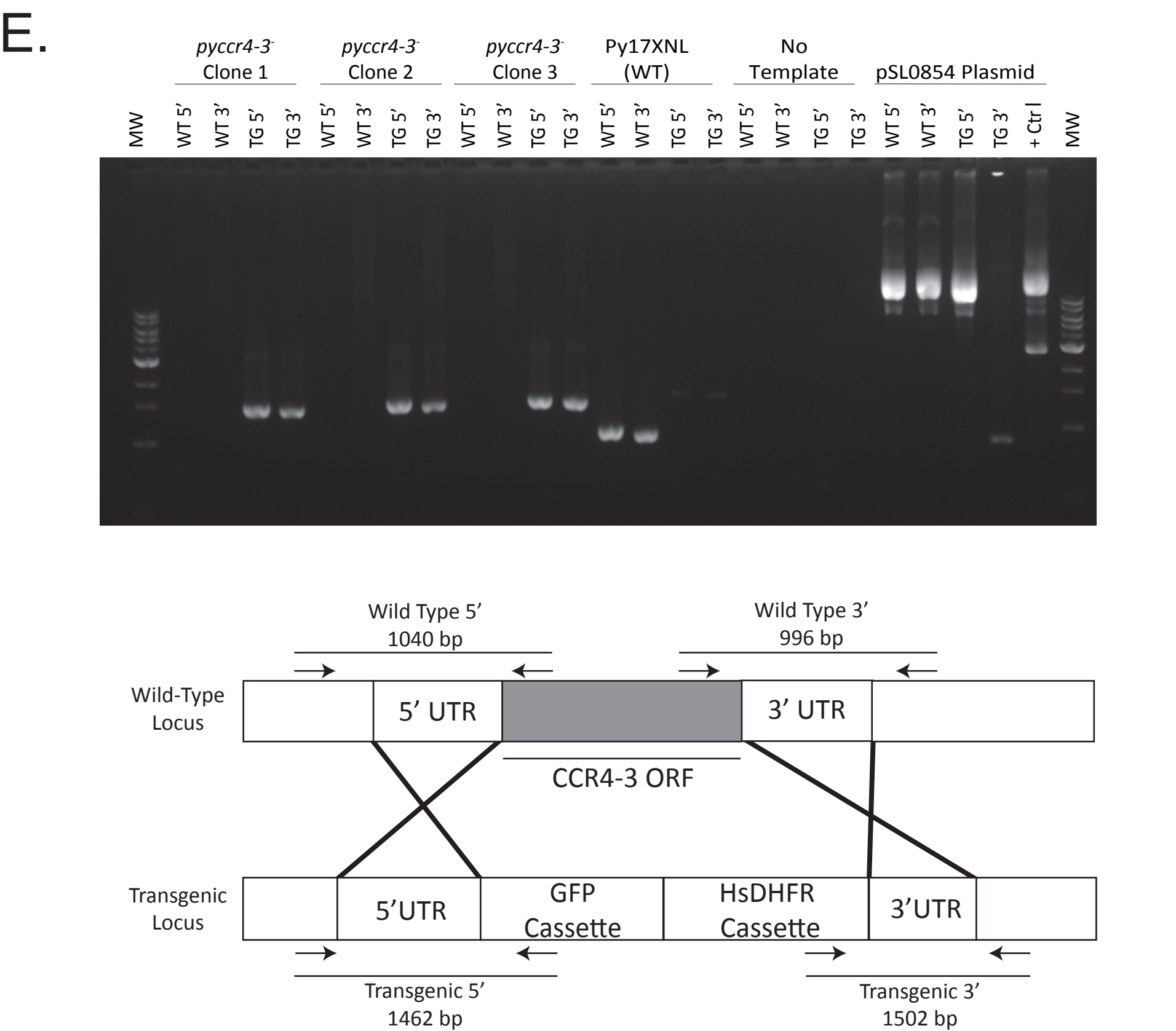

F.

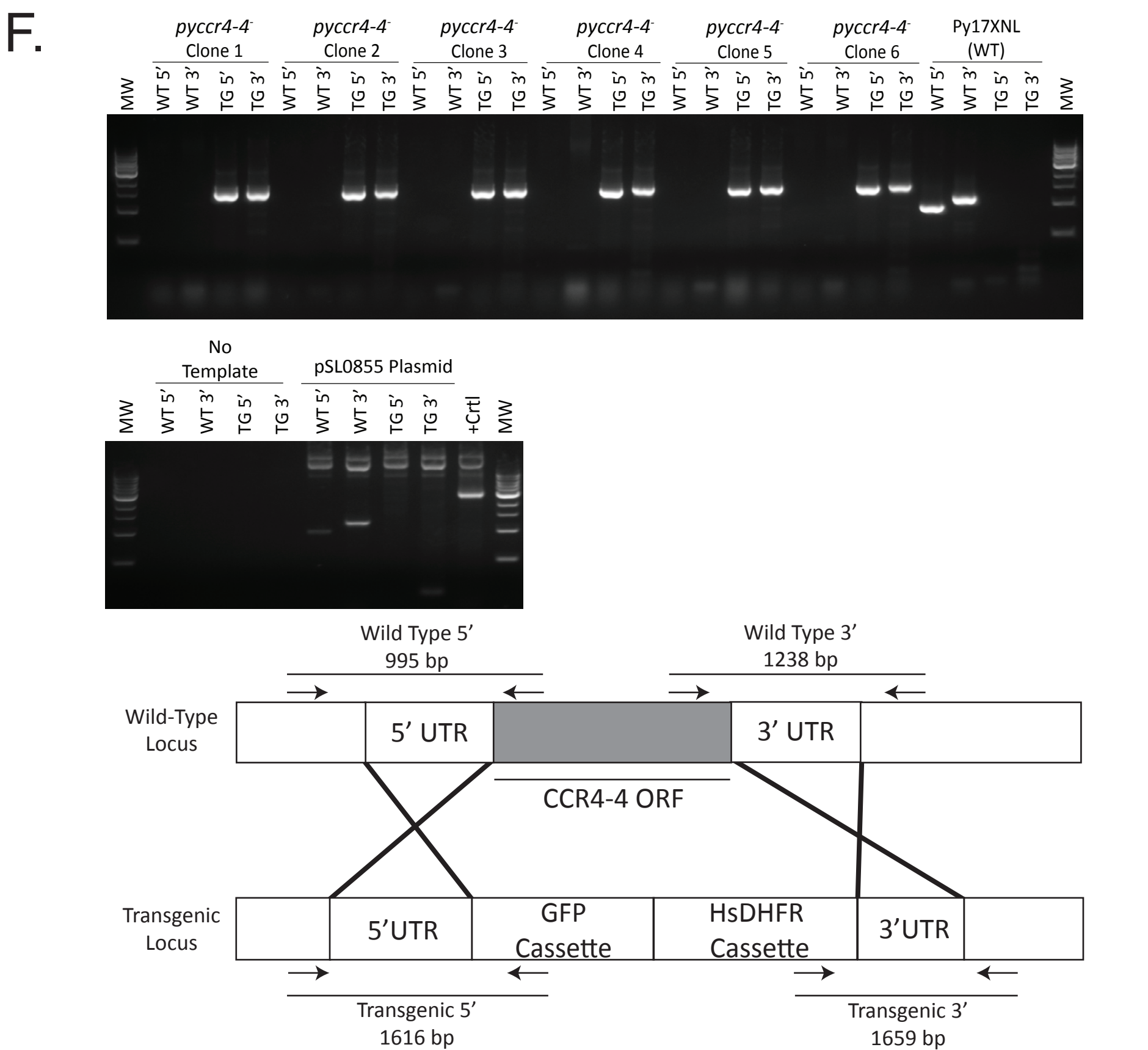

Supplement: S1 Fig — A) Schematics of the four bioinformatically predictable CCR4 domain-containing proteins (CCR4-1, 2, 3, and 4) of Plasmodium species are shown. The four proteins with identified exonuclease-endonuclease-phosphatase domains (shaded white rectangles) are shown to scale with their domain architecture, introns (gaps) and exons (rectangles). Also shown are E-values for their EEP domain based upon their alignment with CCR4 (PLN03144) via the Conserved Protein Domain Database. B) The four bioinformatically predictable CCR4 domain-contains proteins from P. yoelii, the catalytically dead PyCCR4-1 (dPyCCR4-1), CCR4-1 from P. falciparum, and orthologues from S. cerevisiae, human, and mouse were aligned using EMBL Clustal Omega. Shown is the region around the catalytic residues of CCR4. Amino acids noted in red font are the two catalytic residues, while those noted in white font with black highlighting are the two residues that were changed to create dPyCCR4-1. C-F) Genotyping PCR of (C) pyccr4-1-, (D) pyccr4-2-, (E) pyccr4-3-, and (F) pyccr4-4- transgenic parasites. Successful genetic deletions were created using double homologous recombination of the targeting sequence consisting of ~750bp on either side of the ORF. Genotyping was performed by PCR on parasites cloned by limiting dilution using the primers indicated (listed in S7 Table). Independent clones were compared to Py17XNL wild-type control genomic DNA, a no template control, and a plasmid positive control in parallel. (PDF) [file ppat.1007164.s001.pdf]

S2 Figure: Hart *et al.*

A.

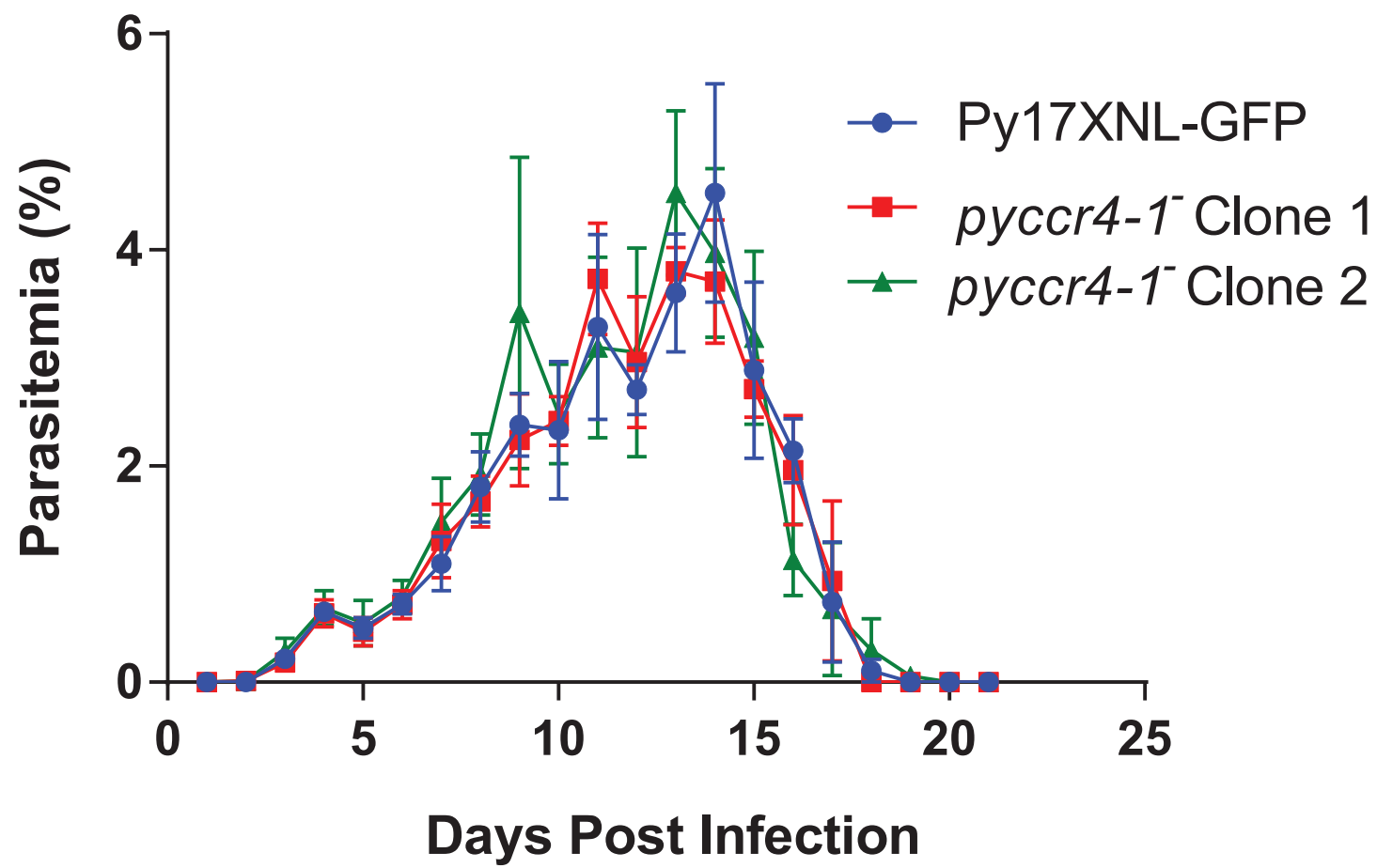

B.

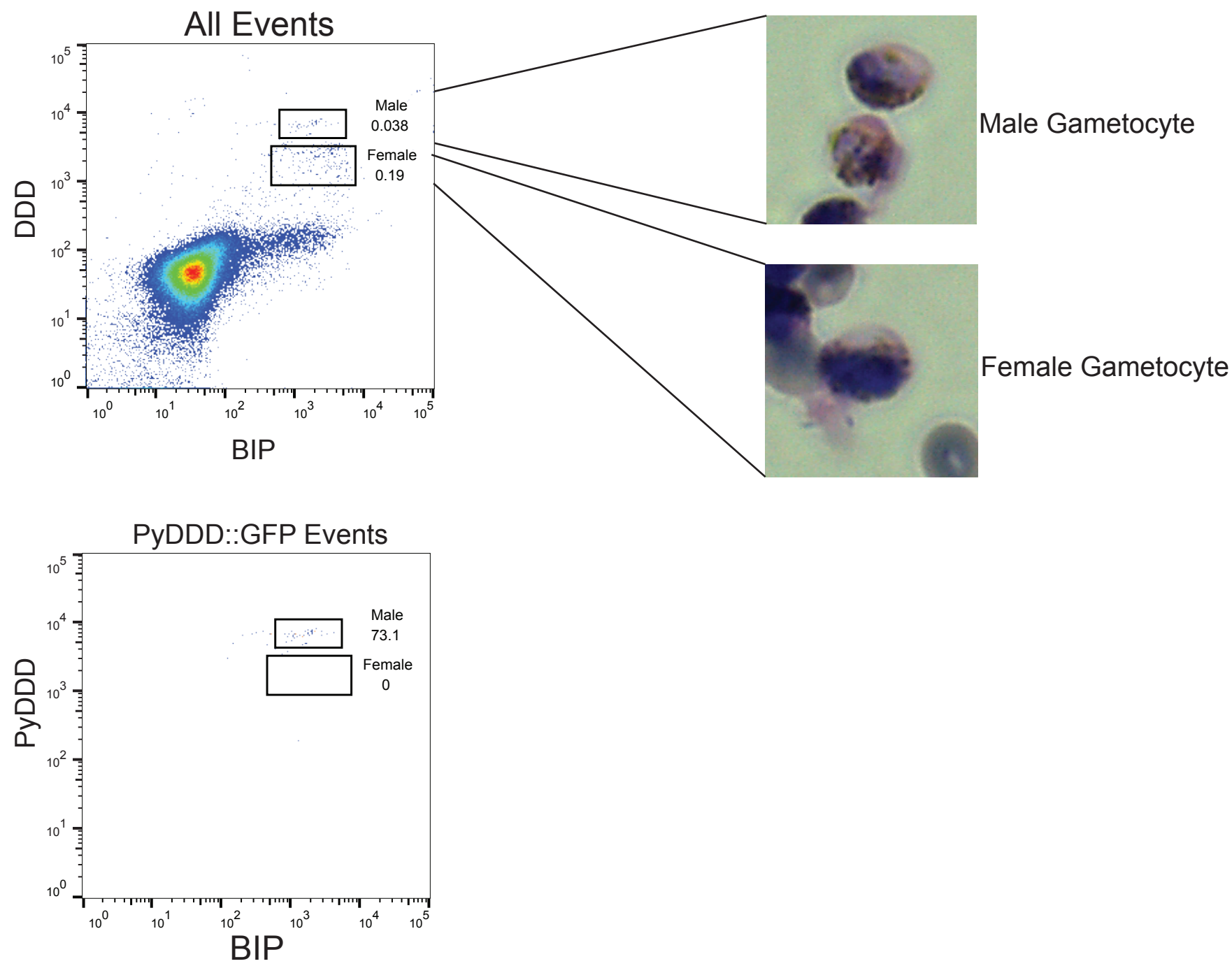

C.

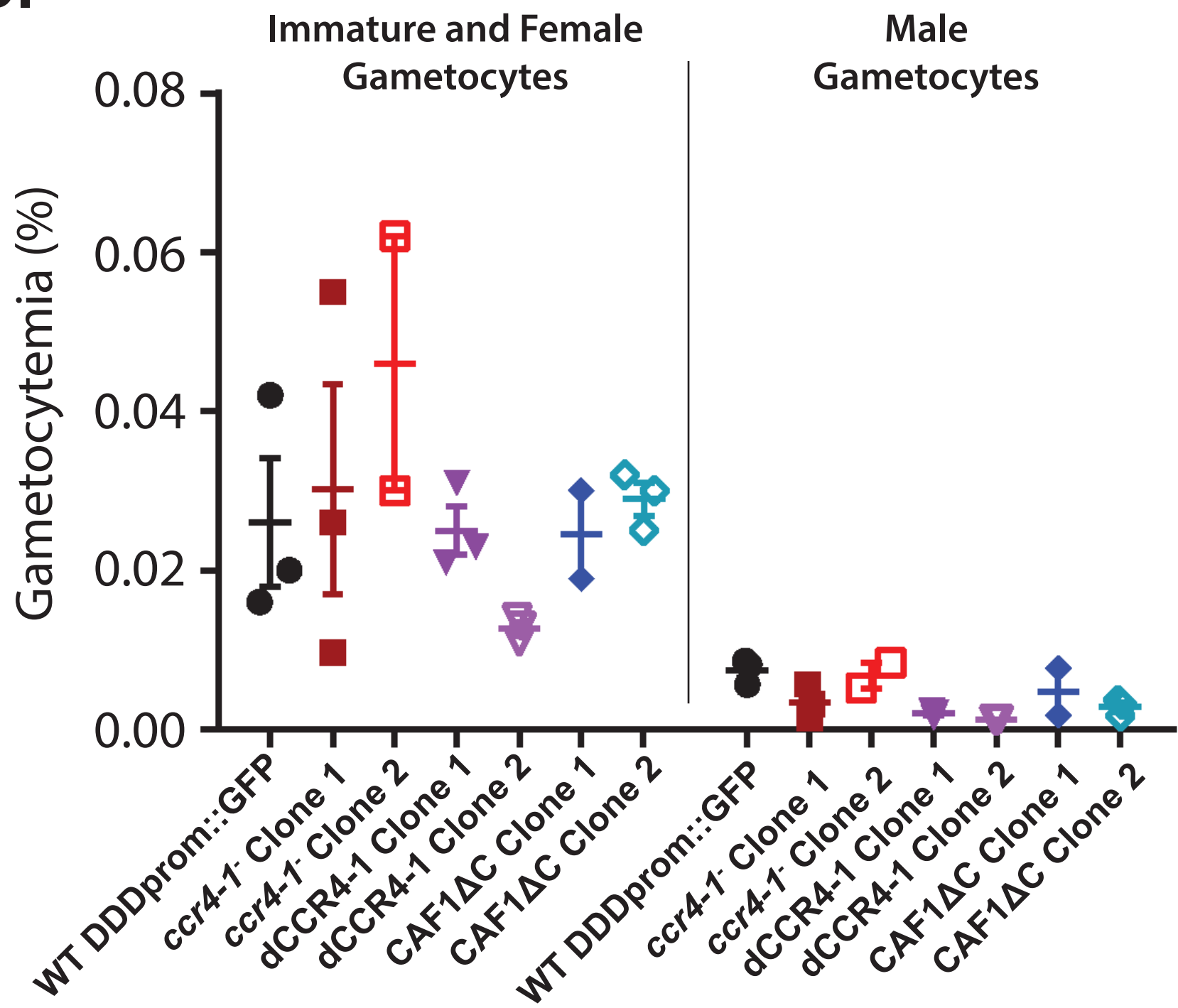

D.

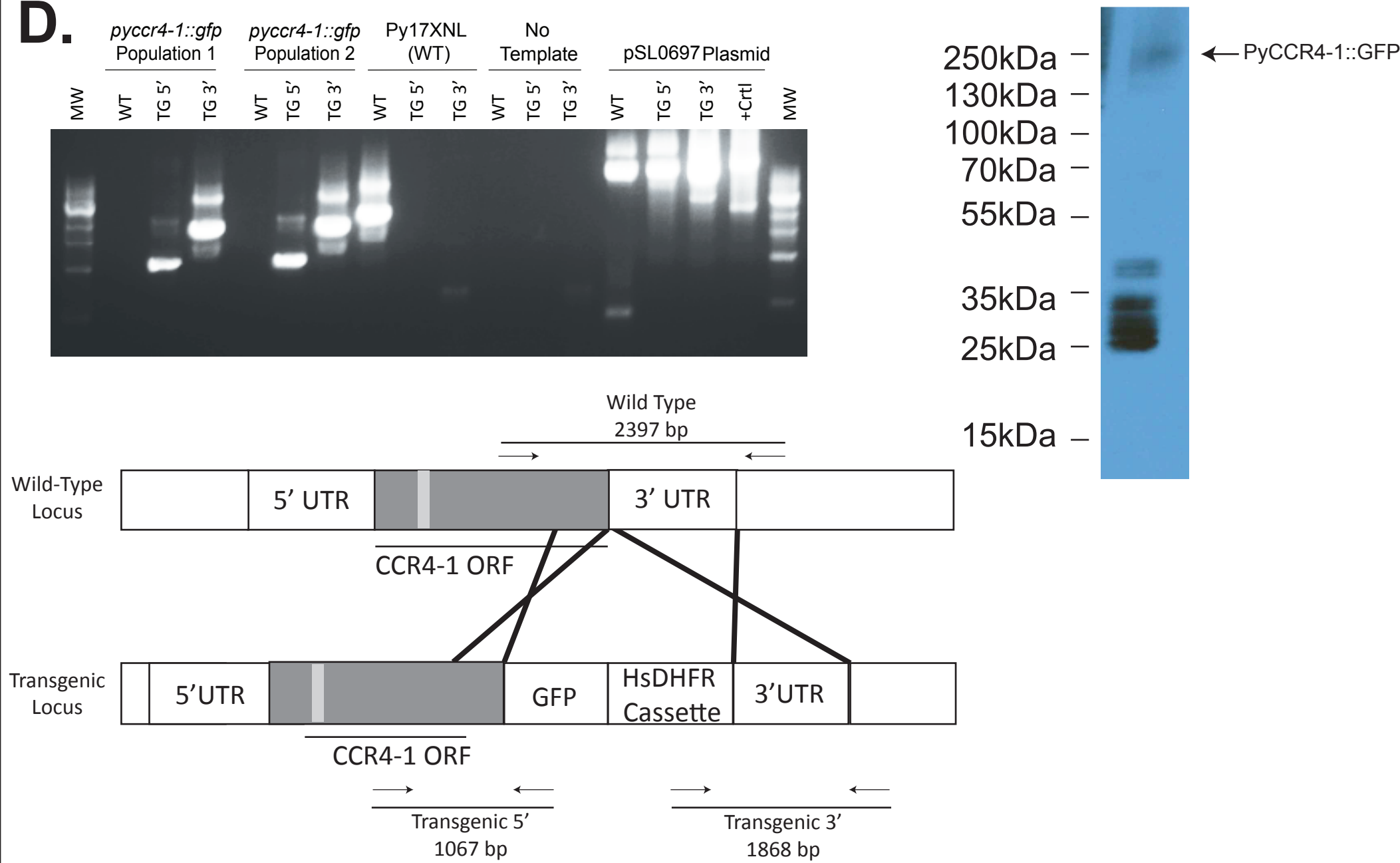

E.

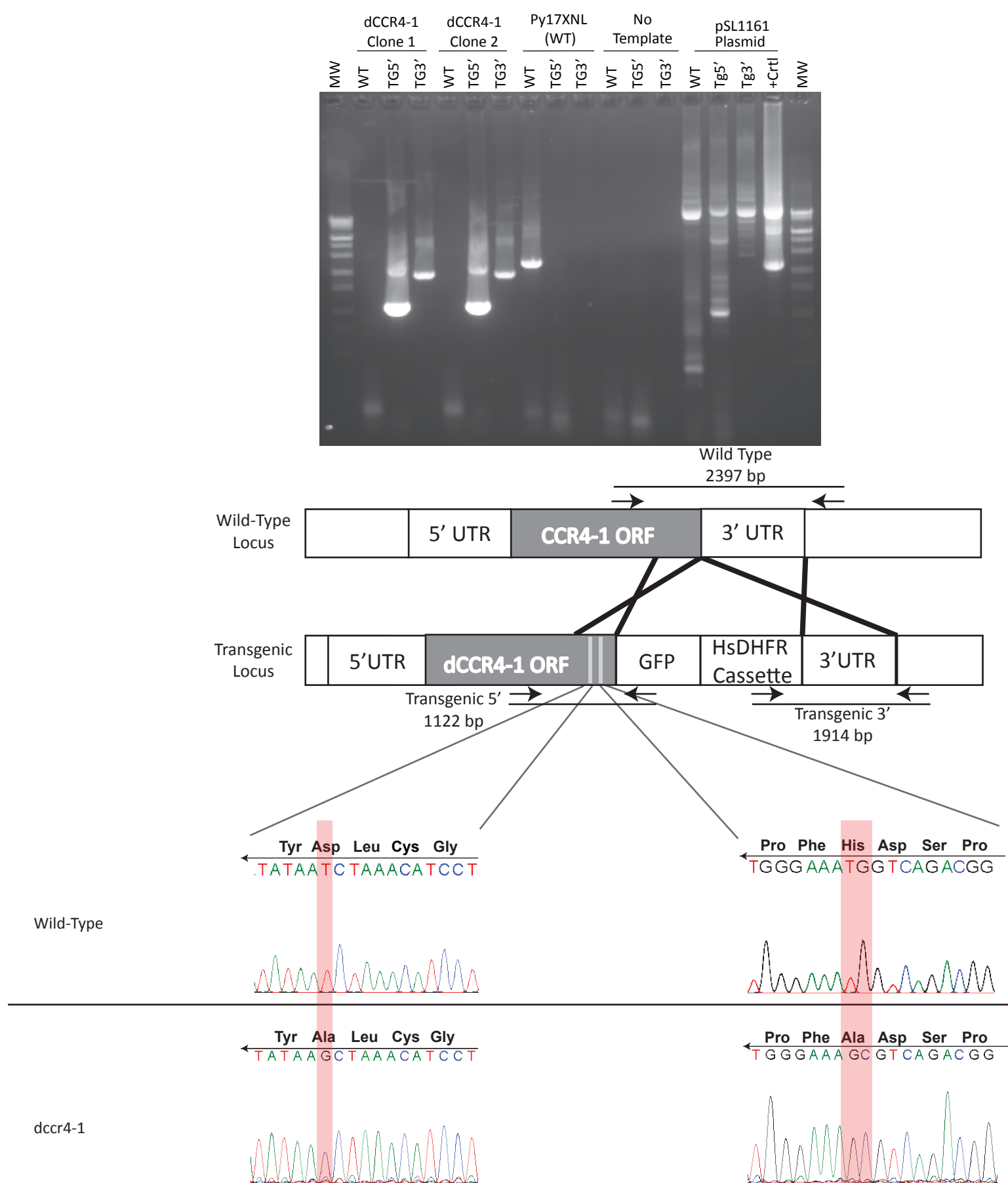

F.

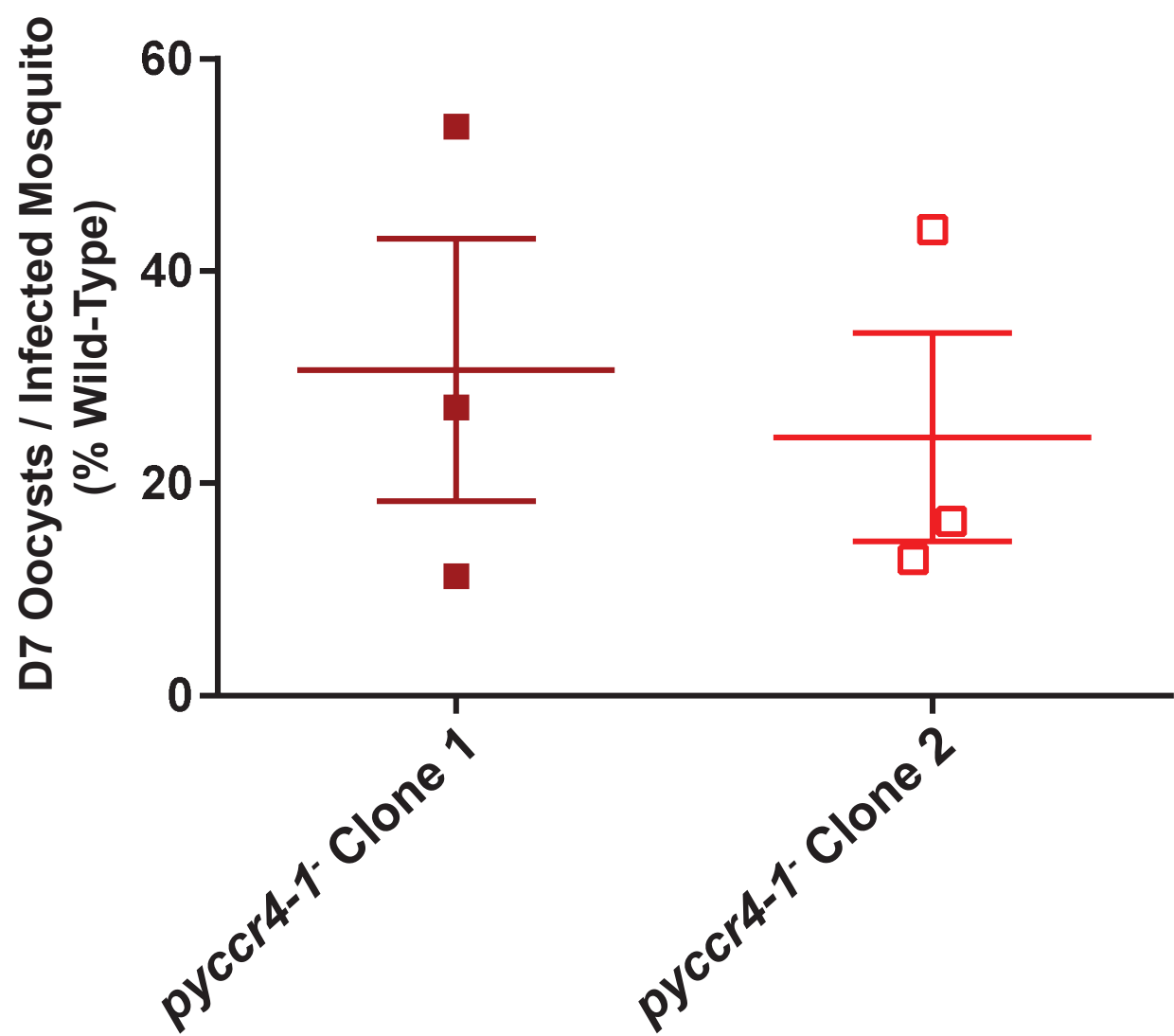

Supplement: S2 Fig — A) Asexual blood stage growth was monitored for two pyccr4-1- transgenic clonal lines compared to a WT-GFP control line over the entire course of an infection. No significant difference in growth kinetics was observed. B) Gametocyte counts were performed using flow cytometry. Asexual stage parasites were removed with two days of sulfadiazine treatment and WBC’s were removed using a cellulose column. PyDDD high and BIP + cells were scored as mature male gametocytes and DDD mid and BIP+ cells were scored as immature or female gametocytes. No red blood cells were excluded in this analysis, and thus permitted measurement of gametocytemia. A PyDDD promoter driving GFP was used to establish gating of mature male gametocytes. PyDDD+ cells were FACS selected and observed to be male gametocytes by Giemsa staining and could undergo gametogenesis (exflagellation assay). C) Mature male or immature/female gametocytemia were counted by flow cytometry for wild-type and transgenic parasite lines in this study. D) Genotyping PCR of pyccr4-1::gfp transgenic parasites was performed by PCR on parasites as described in S1 Fig. Expression of PyCCR4-1::GFP was detected at ~250kDa by western blotting of immunoprecipitated material. E) Genotyping PCR of dPyCCR4-1 transgenic parasites is shown. A successful replacement of the PyCCR4-1 catalytic residues were created using double homologous recombination to insert a C-terminal GFP tag and stop codon following the PyCCR4-1 stop codon. Genotyping was performed by PCR on parasites as described in S1 Fig. Sequencing results are shown demonstrating the appropriate base change to substitute alanine for these two amino acids has occurred. F) Mosquitoes fed upon mice infected with pyccr4-1- parasites performed 2 days after the peak day of exflagellation (D7). The number of oocysts per infected mosquito on day seven post-infectious blood meal are plotted. Data represents at least 20 dissected mosquitoes per biological replicate conducted in triplicate [file ppat.1007164.s002.pdf]

S3 Figure: Hart *et al.*

A.

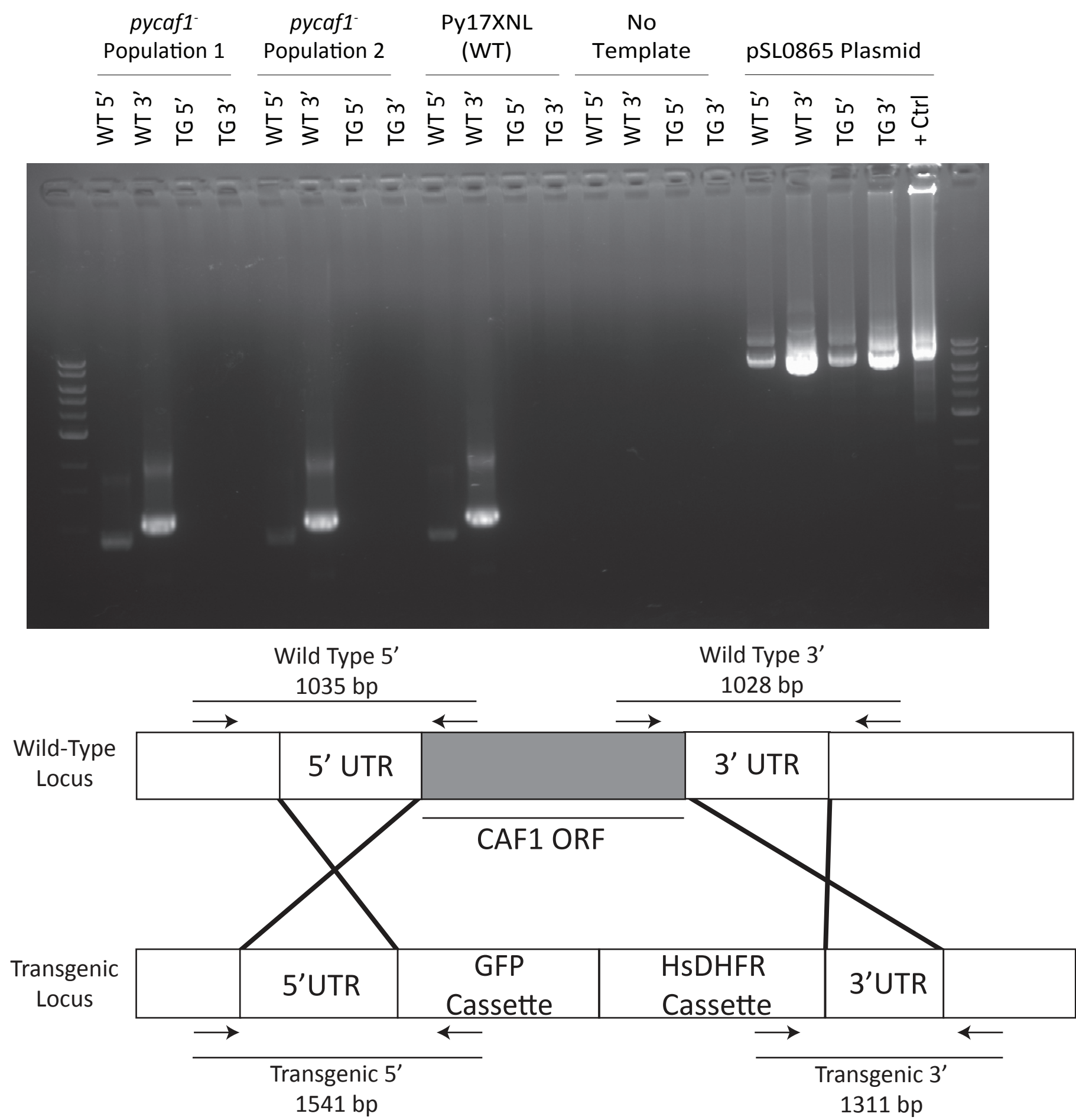

B.

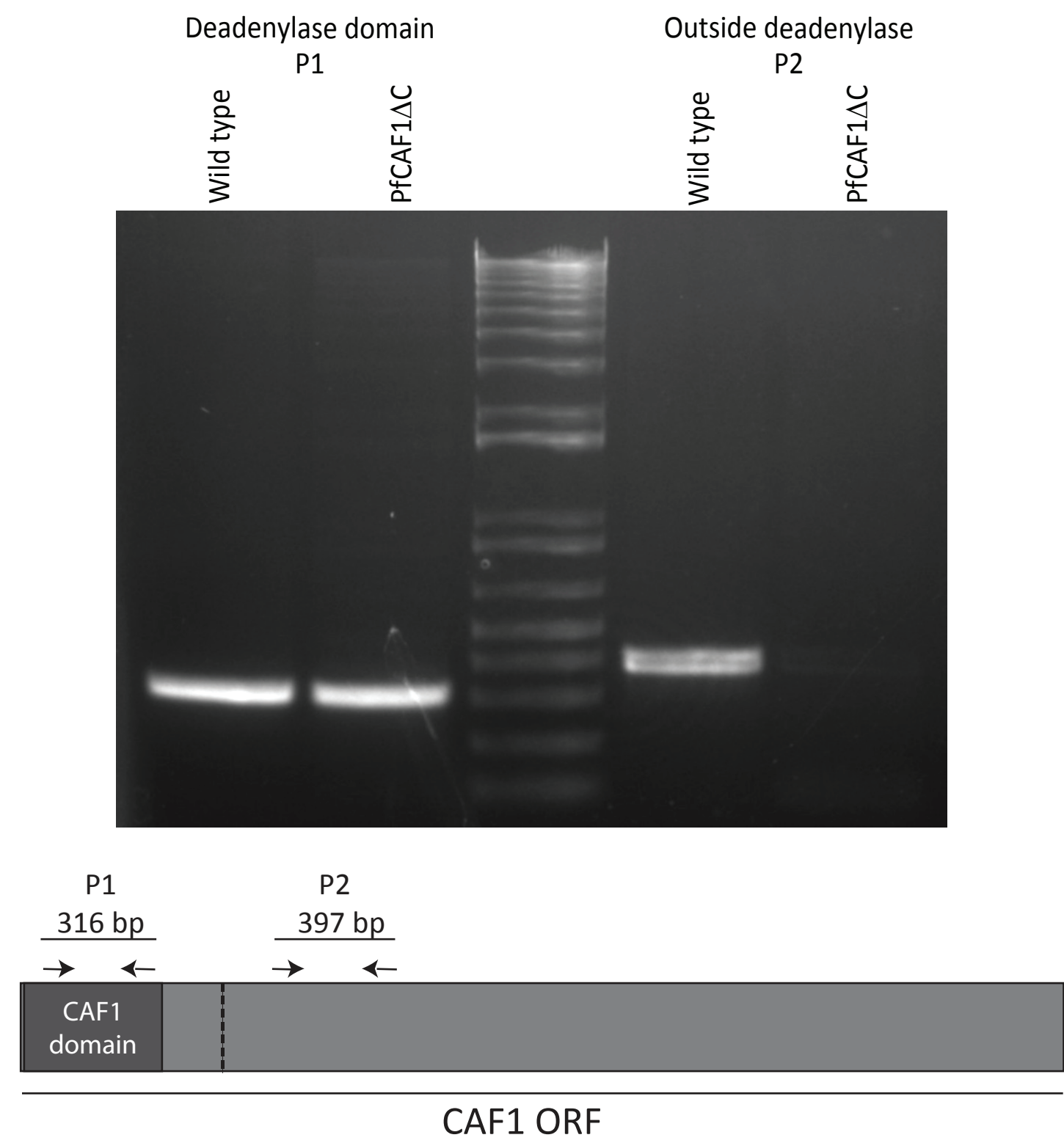

C.

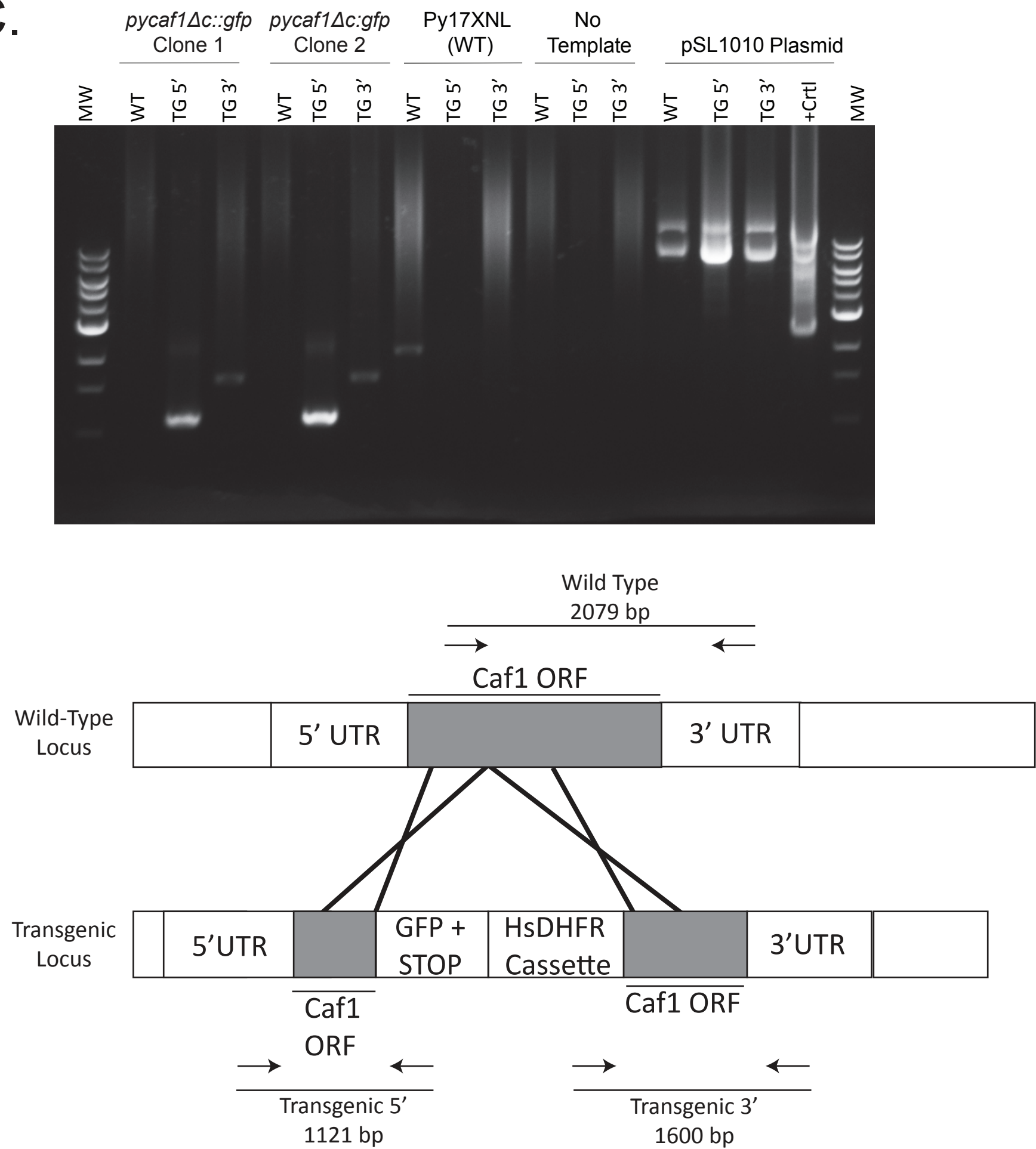

D.

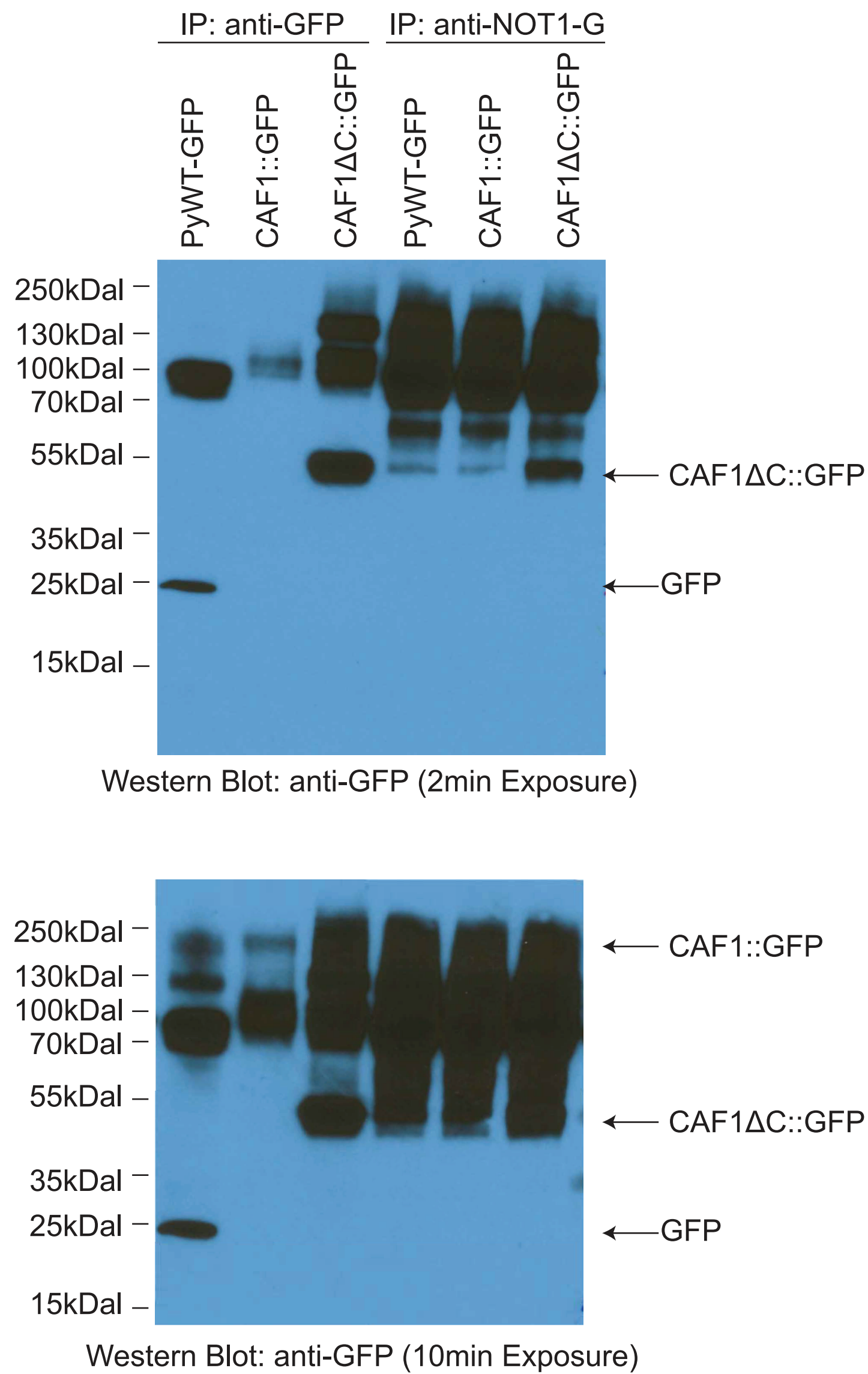

Supplement: S3 Fig — A) Genotyping PCR of pycaf1- transgenic parasites. An attempt at the deletion of pycaf1 by double homologous recombination using targeting sequences consisting of ~750bp on either side of the ORF is depicted. Genotyping was performed by PCR as described in S1 Fig. B) A P. falciparum line carrying a piggyBac transposon inserted after the CAF1 deadenylase domain makes a truncated transcript. A schematic of RT-PCR primers aligned to the CAF1 ORF is provided as a reference, with the site of the piggyBac disruption indicated by a dotted line. C) Genotyping PCR of a pycaf1 disruptant transgenic parasites is shown. A successful disruption of pycaf1 was created using double homologous recombination to insert a C-terminal GFP tag and stop codon following the CAF1 domain (PyCAF1ΔC). Genotyping was performed by PCR as described in S1 Fig. D) Immunoprecipitations were performed on three different parasite backgrounds, PyWT-GFP, PyCAF1::GFP, and PyCAF1ΔC using either an anti-GFP or anti-NOT1-G antibody. These were then probed with a different anti-GFP antibody than the one used for immunoprecipitation. A 2 min exposure and 10 minute exposure are provided to allow visualization of GFPmut2, full length PyCAF1::GFP, and PyCAF1ΔC. (PDF) [file ppat.1007164.s003.pdf]

S4 Figure: Hart *et al.*

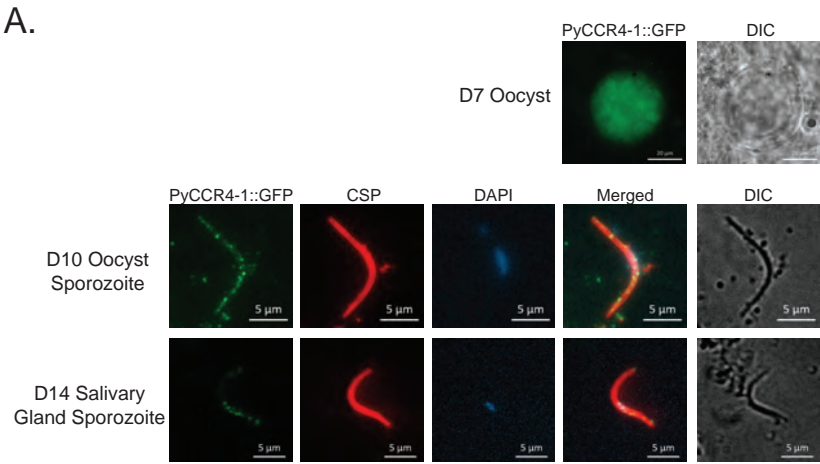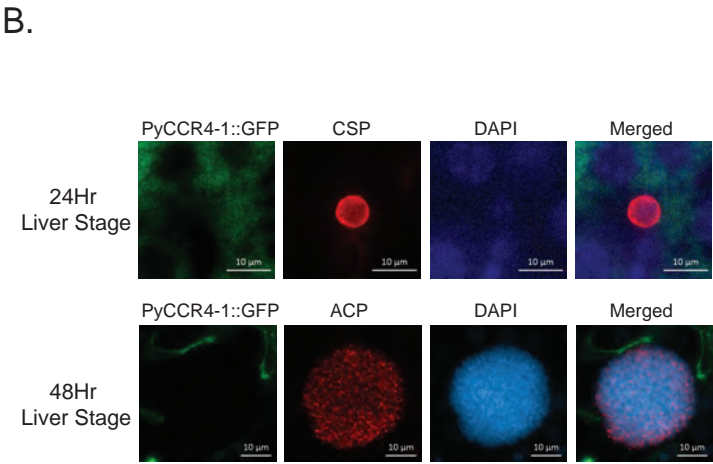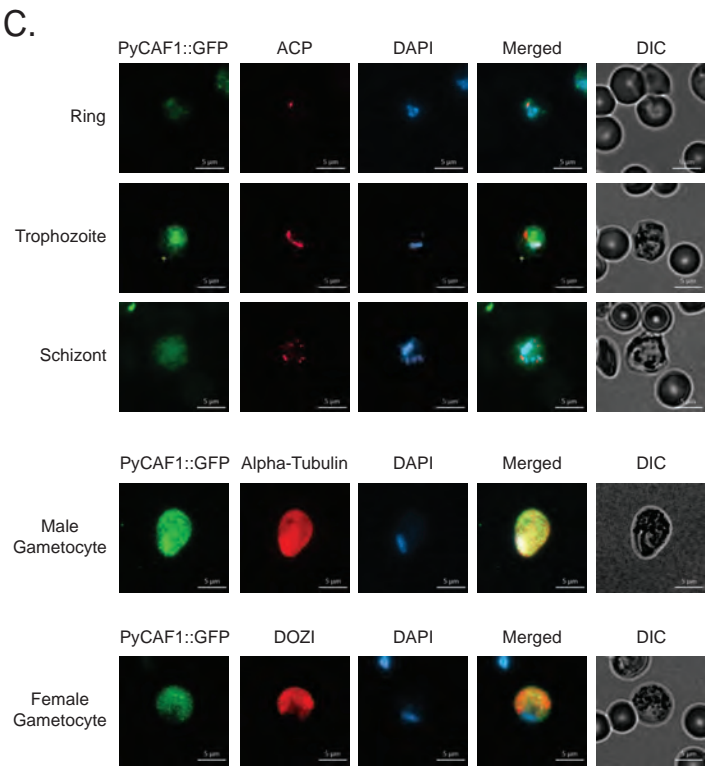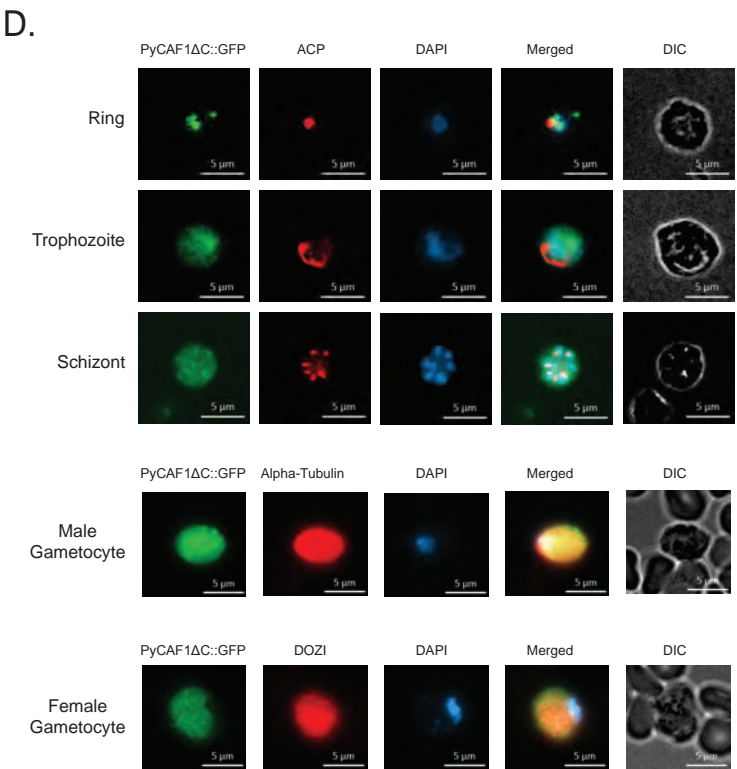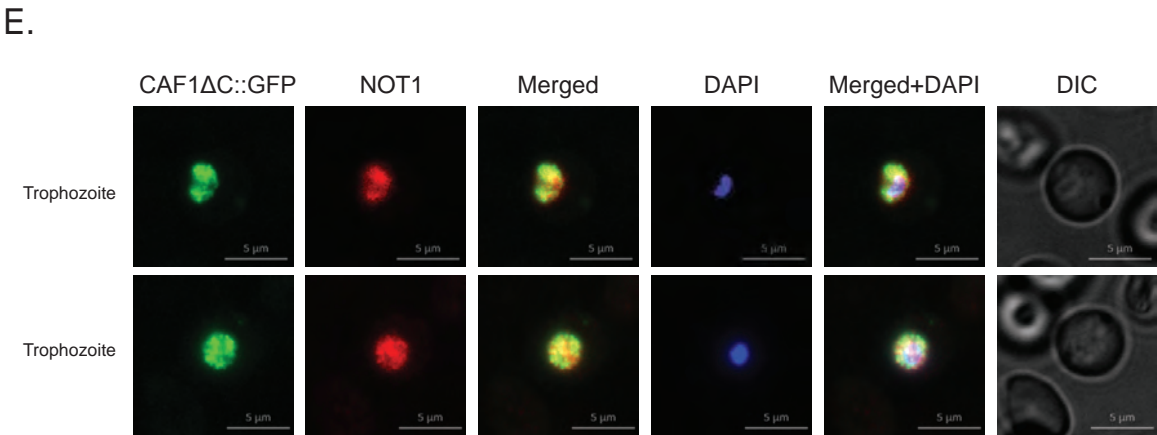

Supplement: S4 Fig — A, B) PyCCR4-1::GFP is expressed in mosquito stage parasites but is not detectable in liver stage parasites. Representative images are shown of A) oocyst sporozoites, salivary gland sporozoites, and B) 24 hour and 48 hour liver stage parasites treated with DAPI and antibodies to GFP (to detect PyCCR4-1::GFP) or to stage-specific cellular markers (CSP, ACP, alpha-tubulin, or DOZI). Oocysts were imaged by live fluorescence. Scale bars are either 20 microns (oocysts), 5 microns (sporozoites), or 10 microns (liver stage parasites). C, D, E) PyCAF1::GFP and PyCAF1ΔC::GFP parasites were imaged by IFA as described in Fig 3 using anti-GFP, anti-ACP, and anti-PyNOT1 antibodies. (PDF) [file ppat.1007164.s004.pdf]

S5 Figure: Hart *et al.*

A.

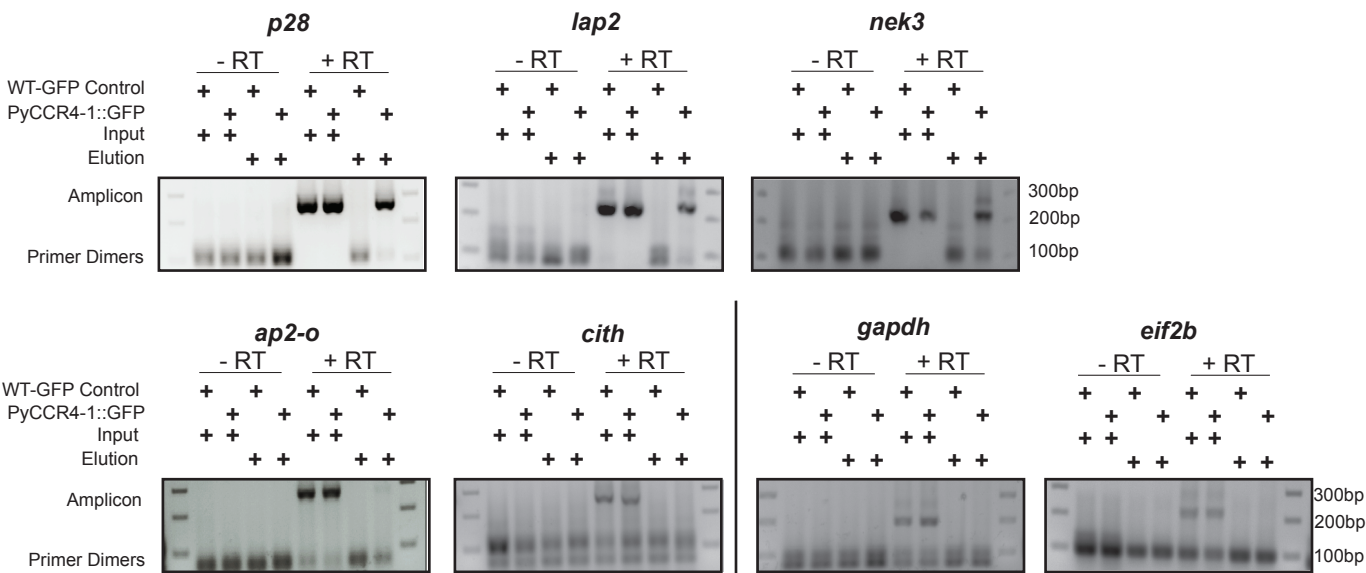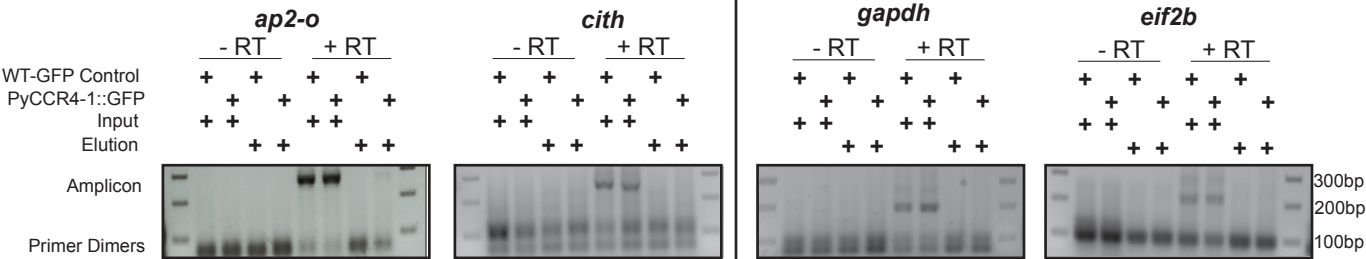

B.

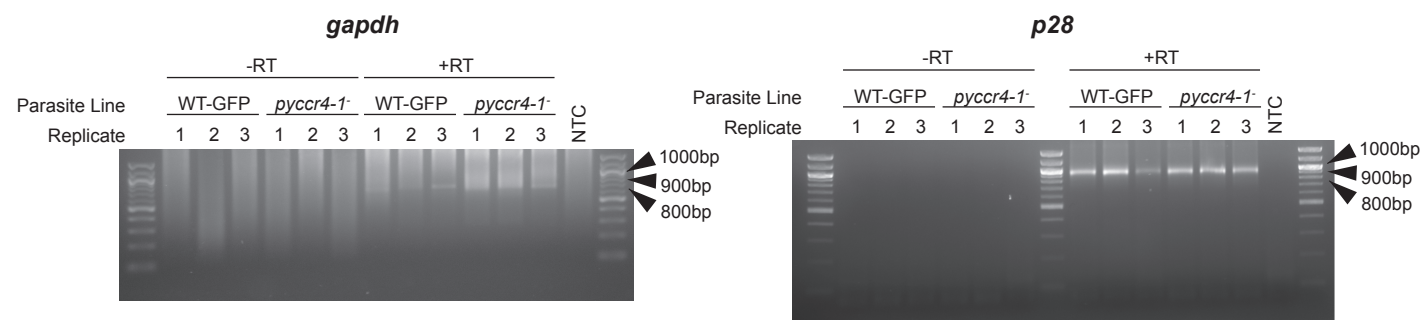

C.

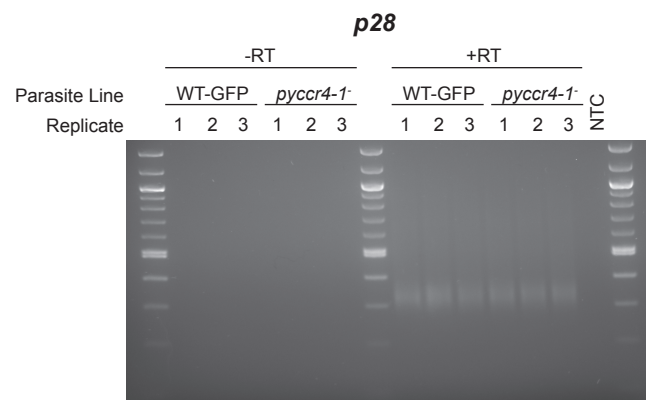

Supplement: S5 Fig — A) Control reactions of samples not treated with reverse transcriptase (-RT) are provided in addition to the +RT experimental samples for all assays. Assessment of gapdh (B) and p28 (B,C) by cRT-PCR is also provided as a control. (PDF) [file ppat.1007164.s005.pdf]
