# Supplementary material for: Plasmodium male gametocyte development and transmission are critically regulated by the two putative deadenylases of the CAF1/CCR4/NOT complex
Source: PLoS Pathog. 2019 Jan 31;15(1):e1007164. doi: 10.1371/journal.ppat.1007164 (PMC6355032; doi:10.1371/journal.ppat.1007164)
Supplement: S6 Fig — Sanger sequencing of cRT-PCR products from the circularized p28 transcript using primers that anneal within the coding sequence (upper case) permitted identification of the 5’ (red font, lower case) and 3’ UTRs (blue font, lower case), as well as the poly(A) tail (black font, lower case, underlined). Sequencing could not extend robustly through the poly(A) tail to provide an exact length from either forward or reverse sequencing primers (denoted by dashes). Sequences of the circularized gene product are provided from a cloned PCR product that is representative of UTRs from both wild-type and pyccr4-1- samples. (PDF) [file ppat.1007164.s006.pdf]

S6 Figure: Hart *et al.*

>*P. yoelii* p28 transcript, cRT-PCR product

GGTACAGGTAGTGGTACTGGAACACCAGCAAATAGTAGTATTATGAACGGAATGTCAATATTCAGCATTA
TTGCATTACTTGTTATTTATATAGCAGTAATGTAAtatatccaattgttatcgcatattgtaggaatatc
tatatagagagagacaaaaaaaaattaaaacagatttgacatttaaataattgatatttgtcgtggctaa
gtttacaagaatgaaataactatttttttttttttttgtctatttaaatatgttaaaaatatgtaaattt
ttttttccgtttaaagggaaaaggaatacataaaaatggcattaatatgatatacaatgaggagaacaag
aggggaaataataaaataaataaaaatatacatttaattgtatatatttttattatgatattattttttc
tcttccaacttgtatttcatttgttgttttcaatatttttttttgttttgttttttttttgttttgtttt
tttcattattccttttaagattaatgacttaaatttaactatattgaaaattaaaaaaaaaaagtataaa
gtataaatagatttataaaaaaaaaaaaaaaaaaaaaaaaaaaaaaaaaaaaa-----------------
aaaaaaaaaaaaaaaaaaaaaaaaaaaaaaaaaaaaaaaaattttgatttcactttatacatttttttaa
atttaacattatttatattctcataatttccgtaaaaaaaacaaaacaatttcataaaattatactcata
acaacagttattttaacaaaaatattttatactaaattttcacgaaaATGAATTTTAAATACAGTTTTAT
TTTTTTATTTTTTATCCAACTTGCGATAAGATATAATAATGCAAAAGTCACTGTAGACACG
