# Supplementary material for: Plasmodium male gametocyte development and transmission are critically regulated by the two putative deadenylases of the CAF1/CCR4/NOT complex
Source: PLoS Pathog. 2019 Jan 31;15(1):e1007164. doi: 10.1371/journal.ppat.1007164 (PMC6355032; doi:10.1371/journal.ppat.1007164)
Supplement: S1 File — (DOCX) [file ppat.1007164.s007.docx]

Extended Materials and Methods

*Ethics Statement*

All animal care strictly followed the Association for Assessment and Accreditation of Laboratory Animal Care (AAALAC) guidelines and was approved by the Pennsylvania State University Institutional Animal Care and Use Committee (IACUC# 42678-01). All procedures involving vertebrate animals were conducted in strict accordance with the recommendations in the Guide for Care and Use of Laboratory Animals of the National Institutes of Health with approved Office for Laboratory Animal Welfare (OLAW) assurance.

Experimental Animals

Six-to-eight week old female Swiss Webster mice from Harlan (recently acquired by Envigo) were used for all of the experiments in this work. *Anopheles stephensi* mosquitoes (obtained from the Center for Infectious Disease Research; Seattle, WA) were reared at 24C and 70% humidity and were used to cycle *Plasmodium yoelii* (17XNL strain) parasites.

Production of Transgenic Parasite Lines

Transgenic *Plasmodium yoelii* (17XNL strain) parasites were created using targeting sequences to incorporate sequence into the target gene using double homologous recombination. Targeting sequences corresponding to ~750 bp regions within and/or flanking the open reading frame of the gene-of-interest were generated by PCR using purified *Plasmodium yoelii* 17XNL genomic DNA using Phusion polymerase (NEB) and specific primers (S7 Table) in order to provide homology regions for gene editing. These PCR products were combined by Sequence Overlap Extension (SOE) PCR to create a final targeting sequence [1]. These sequences were gel extracted (QIAquick Gel Extraction Kit, Qiagen, Cat# 28706), precipitated with ethanol, and ligated into an intermediate vector (pCR-Blunt, Life Technologies) for sequence verification. The targeting sequence was enzymatically digested out of the intermediate vector and inserted into a pDEF final vector, either pSL0444 that contains a Green Fluorescence Protein mutant 2 (GFPmut2) expression cassette for detection and a HsDHFR expression cassette for drug selection for gene deletions, or pSL0442 that contains a C-terminal GFPmut2 tag for detection and a HsDHFR expression cassette for drug selection for the. The promoter/5’UTR sequences (1.5kb) of *P. yoelii* dynein heavy chain delta (“PyDDD”, PY17X_0418900) was similarly PCR amplified and used to drive expression of GFP by insertion into pSL0442 with targeting sequences for the *p230p* safe harbor locus. Plasmid DNA was linearized using a unique restriction site between the 5’ and 3’ targeting sequences and was transfected as previously published [2]. Briefly, the *Plasmodium yoelii* 17XNL strain was grown in mice to 1% parasitemia and cultured *in vitro* to the schizont stage. Parasites were purified by an Accudenz discontinuous gradient and were electroporated in Lonza T-cell solution using program U-033 in the presence of a total of 5-10 ug of linearized plasmid DNA. Transfected parasites were injected intravenously into mice and drug cycling was initiated 1 day post transfection by supplying pyrimethamine (0.007% w/v Fisher Scientific, Cat# ICN19418025) in acidified drinking water for three days. Following this, mice were placed on untreated drinking water and parasites were allowed to reach 1% parasitemia. One hundred microliters of infected blood was transferred to a new mouse, and drug cycling was repeated as above. Upon reaching 1% parasitemia, the mouse was euthanized and the parasites were isolated. Parasite genomic DNA was purified (QIAamp DNA Blood Kit, Qiagen, Cat# 51106) and genotyping PCR was performed to assess the ratio of WT to transgenic parasites present. Clonal parasite populations were produced using limiting dilution cloning by injecting one parasite in 100 ul RPMI media into groups of mice. Mice where <37% of the group became blood stage patent were considered to be infected with a single parasite (as per the Poisson distribution) and genotyping PCR was used to confirm that they are transgenic and clonal. The PfCAF1ΔC line was previously generated as described [3]. Validation of CAF1 transcript expression was performed via RT-PCR on 100ng DNase-treated RNA from PfCAF1ΔC and NF54-control parasites using primer sets supplied in S7 Table.

Production and Accudenz Purification of *P. yoelii* Schizonts and Gametocytes

To produce schizonts in culture, infected Swiss Webster mice were exsanguinated by cardiac puncture and the blood was placed in 5ml complete RPMI (cRPMI) (20% v/v FBS in RMPI 1640 with 14.2 ug/ml final gentamycin). The blood was spun at 200 *xg* for 8 min to remove the serum. Blood was then suspended in 30 ml cRPMI and placed in a plugged T75 flask, gassed with a 5% CO_2_, 10% O, 85% N gas mixture and cultured for 12 hours at 37C on a slight incline while on an orbital shaker at 50-60rpm. Parasites were checked by giemsa-stained thin blood smears to ensure that parasites had developed fully into mature schizonts/gametocytes. Once confirmed, aliquots of 30 ml of culture were underlayed with 10 ml of 17% w/v Accudenz in 5 mM Tris-HCl (pH 7.5@RT), 3 mM KCl, 0.3mM disodium EDTA, 0.4x PBS (without calcium and magnesium) and spun at 200 *xg* for 20 min with no brake [4]. Upon completion of the spin, parasites were collected from the interface between the Accudenz and cRPMI layers, transferred to a fresh conical tube, supplemented with an equal volume of additional cRPMI, and spun for 10 min at 200 *xg*. The supernatant was then removed and the parasite pellet processed for downstream applications.

Gametocytes were produced by treatment of the mice at 1% parasitemia with 10 mg/L sulfadiazine (VWR, Cat# AAA12370-30) in their drinking water for two days prior to exsanguination. The blood was placed in 30 ml of pre-warmed cRPMI to prevent activation of gametocytes and parasites were purified as described above.

*P. falciparum* Gametocyte Production

Gametocyte-producing cultures were established as described previously [5] with some modification. Briefly, starter cultures of wild-type *P. falciparum* NF54 and PfCAF1ΔC were grown to ~5% parasitemia in 25 cm^3^ flasks via standard culture conditions in complete medium [RPMI 1640 medium (Sigma-Aldrich) supplemented with 25 mM HEPES (Sigma-Aldrich), 0.2% D-glucose (Sigma-Aldrich), 200 uM hypoxanthine (Sigma-Aldrich), 0.2% w/v sodium bicarbonate, and 10% v/v heat-inactivated human serum] at 6% hematocrit in a tri-gas incubator (5% CO_2_, 5% O_2_) at 37C . On Day 0, starter cultures were then used to inoculate 75 cm^2^ flasks (15ml culture volume at 6% hematocrit) in technical duplicate for each line at 0.5% parasitemia. Parasites were cultured for 17 days with daily media changes and no fresh addition of blood. Samples were taken to monitor parasite development starting at Day 3 post-infection and then every 48 hours until Day 13 post-infection, determined through Giemsa-stained smears. At seven days post-infection, technical replicate flasks were combined into one flask, which was maintained for the duration of the experiment. Cultures were grown in biological triplicate.

PyDDD Recombinant Protein Expression and Purification

Coding sequences for PyDDD (PY17X_0418900, AA1845-2334) was generated by IDT as a codon-optimized gene block, ligated into an intermediate sequencing vector, and subcloned into an expression vector with N-terminal GST and 6xHis tags (pSL0220). This plasmid was transformed into *E. coli* (BL21 (DE3) pLysS CodonPlus Strain) and protein expression was induced at OD_600_ 0.5 in a 20 liter fermenter at 23C with 0.5mM IPTG for 20 hours. The cells were then pelleted and resuspended in 500ml Low-imidazole buffer (25 mM Tris-Cl [pH 7.5] at room temperature [RT], 500 mM NaCl, 10 mM imidazole, 1 mM dithiothreitol [DTT], 1 mM benzamidine, and 10% v/v glycerol). The solution was then sonicated (4 times, 60% amplitude, 30 second exposure, 50% duty cycle) and then spun at 15,000 *xg* for 30 min at 4C to pellet insoluble material. The lysate was then incubated with 5ml of Ni-NTA agarose resin for 1hr at 4C then washed with 50ml low-imidazole buffer, 20ml of mid-imidazole buffer (25 mM Tris-Cl [pH 7.5] at RT, 500 mM NaCl, 50 mM imidazole, 1 mM DTT, 1 mM benzamidine, and 10% v/v glycerol) and then eluted with 40ml high-imidazole buffer (25 mM Tris-Cl [pH 7.5] at room temperature [RT], 500 mM NaCl, 300 mM imidazole, 1 mM dithiothreitol [DTT], 1 mM benzamidine, and 10% v/v glycerol). The pool was then dialyzed into GST lysis buffer (50 mM Tris-Cl [pH 8.0] at RT, 150 mM NaCl, 1 mM DTT, 1 mM benzamidine, and 10% v/v glycerol) and then incubated with 2ml of glutathione agarose for 1hr at 4C. The resin was washed with 20ml GST lysis buffer and eluted with 30ml GST elution buffer (50 mM Tris-Cl [pH 8.0] at RT, 150 mM NaCl, 1 mM DTT, 1 mM benzamidine, 30mM reduced glutathione, and 10% v/v glycerol). Purity was confirmed to be >90% by SDS-PAGE by Coomassie Blue staining. Antibodies were generated in rabbits (screened for pre-immune sera with minimal background reactivity) by Pocono Rabbit Farm and Laboratory (Canadensis, PA).

Live Fluorescence and IFA Microscopy

CCR4-1 and CAF1 expression in blood stages, oocyst sporozoites, salivary gland sporozoites and liver stages was observed by an indirect immunofluorescence assay (IFA), and expression in day seven oocysts was observed by live fluorescence. All samples for IFA were prepared as previously described [6]. Parasites were stained with the following primary antibodies: rabbit anti-GFP (1:1000, Invitrogen, Cat# A11122; 1:1000, Pocono Rabbit Farm & Laboratory, Custom PAb), rabbit anti-PyACP (1:1000, Pocono Rabbit Farm & Laboratory, Custom PAb), rabbit anti-PyNOT1 (1:1000, Pocono Rabbit Farm & Laboratory, Custom PAb), mouse anti-GFP (1:1000, DSHB, Clone 4C9), rabbit anti-HsDDX6 that cross-reacts with DOZI (1:1000, gift from Joe Reese, Custom PAb), mouse anti-alpha tubulin (Clone B-5-1-2) (1:1000, Sigma, Cat# T5168), and mouse anti-PyCSP (1:1000, Clone 2F6 [7]). Secondary antibodies used for all stages were Alexa Fluor-conjugated (AF488, AF594) and specific to rabbit or mouse (1:1000, Invitrogen, Cat# A11001, A11005, A11008, A11012). 4′,6-diamidino-2-phenylindole (DAPI) was used to stain nucleic acids following washing away unbound secondary antibodies and samples were covered with VectaShield anti-fade reagent (Vector Laboratories, VWR, Cat# 101098-048) and a coverslip, then sealed with nail polish prior to visualization. Fluorescence and DIC images were taken using a Zeiss fluorescence/phase contrast microscope (Zeiss Axioscope A1 with 8-bit AxioCam ICc1 camera) using a 40X or 100X oil objective and processed by Zen imaging software.

Measurement of Blood Stage Growth Kinetics

Cryopreserved blood infected with either wild-type ((Py17XNL), *ccr4-1^-^,* dCCR4-1, or CAF1ΔC parasites were injected intraperitoneally into Swiss Webster starter mice and parasitemia was allowed to increase to 1%. This blood was extracted via cardiac puncture and diluted in RPMI to 10,000 parasites per 100 ul (CCR4-1, CAF1ΔC) or 1,000 parasite per 100ul microliter (dCCR4-1, CAF1ΔC). One hundred microliters was injected intravenously (IV) into three mice per replicate for each parasite line. Three biological replicates were conducted, each with three technical replicates. Parasitemia was measured daily by giemsa-stained thin blood smears. Centers of movement/exflagellation centers were also measured daily via wet mount of the blood incubated at room temperature for 10 min by counting the number of exflagellating male gametocytes in a confluent monolayer per 400x field (40x objective x 10x eyepiece).

*P. falciparum* ring-stage parasitemia and total gametocytemia were calculated every two days starting on Day 3 post-infection by averaging counts in 10,000 RBCs across a minimum of two biological replicates (provided in S6 Table). Sexual conversion was calculated as described previously [8] by taking the stage II-gametocytemia on Day T and dividing by ring-stage parasitemia on Day T-2. Samples were taken for exflagellation assays on days 13, 14, 15, and 16 post-infection. Two-hundred microliter samples were taken from each flask and spun down at 0.3 *xg* for 30 seconds. Supernatant was removed and a 20 ul aliquot of remaining blood pellet was mixed with 20 ul of heat-inactivated human serum previously warmed to 37°C. The mixture was then allowed to incubate at room temperature for 15 min, after which exflagellation events were counted under 40x magnification for 10 fields-of-view.

Flow Cytometry Gametocyte Counts

Cryopreserved blood infected with either wild- type (Py17XNL), *ccr4-1^-^*, dCCR4-1, or CAF1ΔC parasites was injected intraperitoneally into starter mice and transferred as above (1000 parasites/100ul). On Day 5, gametocytes were produced by treatment of the mice with 10 mg/L sulfadiazine (VWR, Cat# AAA12370-30) in their drinking water for two days. The blood was then placed in 10 ml of pre-warmed cRPMI to prevent activation of gametocytes and spun at 37°C. Blood was then fixed, passed through a cellulose column and stained as described above for IFA. Parasites were stained with the following primary antibodies: mouse anti-PvBIP Clone 7C6B4 (1:1000; [9]) and rabbit anti-PyDynein Heavy Chain Delta Domain (“PyDDD”, PY17X_0418900 AA: 1845 to 2335)) (1:1000, Pocono Rabbit Farm & Laboratory, Custom PAb), along with goat anti-mouse conjugated to AF594 (Fisher Scientific, A11012) and goat anti-rabbit conjugated to AF647 (Fisher Scientific, PIA32733) secondary antibodies. These were then analyzed on a LSR Fortessa (BD) in tube mode and collected samples were analyzed in FlowJo.

Mosquito Transmission Studies

Cryopreserved blood infected with either wild-type (Py17XNL), *ccr4-1^-^*, dCCR4-1, or CAF1ΔC parasites was injected intraperitoneally into starter mice and transferred as above. Centers of movement were checked daily as above and mice were fed to mosquitoes on the peak day of exflagellation (day 5). Mice were anesthetized by IP injection of a ketamine/xylazine cocktail (100 mg/kg ketamine, 10 mg/kg xylazine in sterile 1xPBS without Calcium and Magnesium) and 200 mosquitoes were allowed to feed on two mice per cage for 15 min. The positions of the mice were switched every 5 min to allow for more even feeding. Mosquito midguts were dissected at D7 post feed and analyzed for the prevalence of infection and oocyst numbers by microscopy. Mosquito midguts were dissected on day 10 and their midguts ground and oocyst sporozoite numbers counted on a Hausser Bright-Line Phase hemocytometer (Fisher Scientific, Cat# 02-671-6). Similarly, day 14 mosquito salivary glands were dissected and ground, and salivary gland sporozoite numbers were similarly counted.

Immunoprecipitations, Western Blotting, and Mass Spectrometric Proteomics

Parasite pellets (schizonts) were crosslinked in 1% v/v formaldehyde and lysed using a combination of RIPA lysis buffer (50 mM Tris-HCl (pH 8.0@RT), 0.1% w/v SDS, 1 mM EDTA, 150 mM NaCl, 1% v/v NP40, 0.5% w/v sodium deoxycholate) with a 1x protease inhibitor cocktail (Roche, VWR, Cat# PI88266) and 0.5% v/v SUPERase In (Life Technologies, Cat# AM2694)) for one hour, dounce homogenization (tight) for 30 seconds, and sonication using a single pulse 0.5s at 10% amplitude with a Branson Model 102C Sonicator fitted with the microtip. The parasite lysate was then precleared using streptavidin-coated dynabeads (Life Technologies, Cat# 65601) for one hour at 4C with rotation. Flow through material that did not bind non-specifically to the beads was immunoprecipitated using a biotin-conjugated anti-GFP antibody (Abcam, Cat# ab6658) attached to the streptavidin-coated dynabeads for three hours at 4C with rotation. The beads were washed with modified RIPA wash buffer (50 mM Tris-HCl (pH 8.0@RT), 1 mM EDTA, 150 mM NaCl, 1% v/v NP40) once and then transferred to a new tube. The beads were washed 3 more times with modified RIPA wash buffer and then eluted using elution buffer (50 mM Tris-HCl (pH 6.8@RT), 5% w/v SDS, 5% v/v glycerol, 0.16% w/v bromophenol blue, 200 mM NaCl , 5% v/v β-mercaptoethanol added just before use) at 45C overnight in a heat block. To determine if the pull down was successful, one quarter of the eluted material was subjected to western blotting. Samples were electrophoresed through a 4% stacking and a 10% resolving sodium dodecyl sulfate polyacrylamide gel layers at 200 V for 40 min, and then transferred to a PVDF membrane overnight at 40 V at 4C. Membranes were blocked for two hours with 5% w/v dried milk suspended in 1x PBS and 0.1% v/v Tween-20 and then were probed with a rabbit anti-GFP primary antibody (1:1000, Invitrogen, Cat# A11122), washed with the milk blocking solution, and then were probed with an anti-rabbit secondary antibody (1:1000, Invitrogen, Cat# A16104) conjugated to horseradish peroxidase. SuperSignal West Pico chemiluminescent substrate (VWR, Cat# PI34080) was used for detection by exposure to x-ray film. For mass spectrometric identification of proteins bound to the protein-of-interest, the remaining three quarters of the proteins eluted from the beads were electrophoresed one third of the way through a commercially prepared 4-20% gradient polyacrylamide gel (ThermoScientific, Cat# PI25204). Proteins were stained with Imperial Stain (Fisher Scientific, Cat# PI-24615) for one hour and then repeatedly destained in dH2O. Each lane was cut into four equal slices, and was diced into ~ 1 mm^3^ pieces. The slices were destained further in 300 ul 50 mM ammonium bicarbonate (AmBic) and 50% v/v Acetonitrile (ACN) solution by washing them three times for 10 min each round. The gel slices were then dehydrated in 300 ul 100% ACN and the supernatant was aspirated and residual supernatant was evaporated in a speed vac for 10 min (no heat, no pulsed vacuum). Disulfide bonds were reduced by addition 300 ul DTT to 10mM (final concentration) and incubation at 37C in a thermomixer at 800 rpm for 30 min. The cysteine residues were then alkylated by addition 300 ul 50 mM iodoacetamide and incubation at 37C in a thermomixer at 800 rpm for 30 min. The gel slices were then washed with 300 ul 50 mM AmBic, 50% v/v ACN twice for 10 min each round, and then dehydrated again in 300 ul 100% ACN and dried in a speed vac as above. Digestion with trypsin was performed overnight by addition 50-75 ul 6 ng/ul trypsin gold (Thermo Cat# 90055) in 50 mM AmBic at 37C in a thermomixer at 700 rpm. Supernatant, containing tryptic peptides, was transferred to a fresh microfuge tube. Peptides remaining in the gel slices were extracted at 37C in a thermomixer at 800 rpm for 30 min first with 100 ul 1% v/v formic acid and 2% v/v ACN in nuclease-free water, secondly with 0.5% v/v formic acid and 60% v/v ACN for 30 mins, and finally with 100% ACN. Supernatants containing peptides from all extractions were combined with the supernatant from the tryptic digestion in a single microfuge tube for final drying in the Speed vac. Dried peptides were then submitted to the Harvard Proteomics Core for nano LC/MS/MS analysis. For each sample, peptides were resuspended in 0.1% v/v formic acid and a portion was loaded onto an Acclaim PepMap100 trapping column (column (100 μm × 2 cm, C18, 5 μm, 100 Å, Thermo) at a flow rate of 20 ul/min using 4% v/v aqueous acetonitrile (ACN), 0.1% v/v formic acid (FA) in dH_2_O as a mobile phase. The peptides were separated on an Acclaim PepMap RSLC column (75 um × 15 cm, C18, 2 um, 100 Å, Thermo) with a 90 min 4% - 60% v/v linear gradient of acetonitrile in dH2O containing 0.1% v/v formic acid. The gradient was delivered to the column by a Dionex Ultimate 3000 nano-LC system (Thermo) at 300 nL/min. An LTQ Orbitrap Velos mass spectrometer (Thermo) was set up for a ‘2nd Order Double Play’ type of experiment with the following parameters: full positive-ion 1000-ms FT MS scan at R 60,000 over 350 – 1700 m/z followed by ten ion-trap MS2 scans on most intense precursors with collision-induced dissociation (CID) activation. Precursor ion signal threshold was set at 5000, isolation width 2 m/z, normalized collision energy 35.0 V, activation Q 0.250, activation time 10.0 ms. The precursors were selected using an FT master scan preview mode with charge states less than +2 rejected and monoisotopic precursor selection enabled. Dynamic exclusion repeat duration was 25 s, exclusion duration 13 s, exclusion list size was 200, and the exclusion mass width was +/- 10 ppm relative to the excluded m/z. Polysiloxane signal (m/z 445.1200) was used as a lock mass. The data was processed using the Trans-Proteomic Pipeline (TPP) [10] as described previously with few modifications [7]. Raw data were converted to .mzml format using msconvert [11] and searched using both X!Tandem [12] and Comet [13]. Spectra were searched against reference sequences downloaded in February 2016 from *Plasmodium yoelii* 17X (PlasmoDB, v27), mouse (Uniprot), and common contaminants (Common repository of adventitious protein sequences, [14] and randomized decoys generated through TPP. iX!Tandem and Comet searches were combined in iProphet [15] and protein identifications were determined by Peptide Prophet. Only proteins with a highly stringent false positive error rate of less than 1% are reported. To combine replicate proteomics datasets, SAINT version 2.5.0 was used [16]. The algorithm utilizes the total spectral abundance for each protein and the protein length to normalize and determine the probability of interaction with the bait (CCR4-1::GFP, CAF1ΔC::GFP). The algorithm was run with the following settings: lowMode=1, minFold=0, normalize=1. Only proteins with SAINT scores below 0.1 (most stringent) or 0.35 (stringent) were considered significant hits and included in the analyses, as used previously [17-19].

Total Proteomics by Mass Spectrometry

Mixed blood-stage parasites from one mouse infected with *P. yoelii* wild-type (Py17XNL strain) (3.95% parasitemia) or *ccr4-1^-^* (5.85% parasitemia) transgenic parasites were purified by cellulose column purification (to remove WBC’s) and saponin lysis followed by a 4000 *xg* spin for 10 min. The pellets were washed with 1xPBS and spun again same as before. Parasite pellets were placed in SDS-PAGE sample buffer. Samples were then sonicated with a Branson Model 102C Sonicator fitted with the microtip (10% amplitude; 50% duty cycle; 10 bursts) three times allowing the sample to cool and were spun to remove bubbles between each round. Beta-mercaptoethanol was added to a final concentration of 5% to reduce disulfide bonds. Samples were then heated to 70 C for 5 min and loaded on a commercial 4-20% gradient polyacrylamide gel (Thermo, Cat# PI25204). Samples were electrophoresed until the dye front reached near the bottom of the gel, and individual sample lanes were separated and stained with Imperial Stain (Thermo Scientific, Cat#: 24615) for 1.5 hours and then destained for 1.5 hours in dH2O. Each lane was cut into 24 separate slices and each slice was submitted for independent mass spectrometric analysis at the Penn State Mass Spectrometry Core using the same run parameters as described above for proteomic analyses conducted at the Harvard Proteomic Core.

Total and Comparative RNA-seq

Gametocyte samples were collected as described above. Following purification by an Accudenz gradient, mouse RBCs were subjected to lysis with 0.1% w/v saponin in 1xPBS, then washed with 1xPBS. Released parasites were then lysed immediately. RNA from the samples was isolated by the QIAgen RNeasy Kit (QIAgen, Cat No. 74104) using the manufacturer’s protocol with the additional on-column DNaseI digestion. RNA yields were quantified spectrophotometrically (NanoDrop 2000c, Thermo Scientific), and RNA samples were submitted to the Penn State Genomics Core Facility. The quality of all samples was confirmed by measuring RNA Integrity Number (RIN) using the Agilent Bioanalzyer. A barcoded library was made from each sample by using the Illumina TruSeq Stranded mRNA Library Prep Kit (Illumina, Cat# RS-122-2101) according to the manufacturer's protocol. Quantitative PCR (qPCR) was performed to determine the concentration of each library and an equimolar pool was made of all libraries. The library pool was sequenced on an Illumina HiSeq 2500 in Rapid Run mode according to the manufacturer's protocol. For gametocyte samples, 100 nt single read sequencing was performed. The resulting data was mapped to the *P. yoelii* 17XNL strain reference genome (plasmodb.org, v32 using Tophat2 in a local Galaxy instance (version .9). Gene and transcript expression profiles for both WT-GFP and *ccr4-1^-^* assemblies were generated using htseq-count (Galaxy version 0.6.1galaxy3) [20] using the union mode for read overlaps. Count files were merged and compared using DESeq2 (Galaxy version 2.11.39 [21]). Six biological replicates were used for the WT transcriptomic profile, while four replicates were used in for the *ccr4-1^-^* profiles. These were analyzed by a mean fit type with outlier replacement turned on to normalize the variance between the count files. The P-adjusted value was used for all analyses.

Circular Reverse Transcription PCR (cRT-PCR)

RNA was isolated from purified *P. yoelii* wild type or *pyccr4-1^-^* gametocytes by TRIzol/chloroform extraction and extensive DNaseI digestion. The 7-methylguanosine cap was removed from 10ug of total RNA using 2.5U Cap-Clip Acid Pyrophoshatase (CellScript, Madison, WI, #C-CC15011H) in 1xCap-Clip Buffer supplemented with 10U Murine RNase Inhibitor (NEB, #M0314) at 37C for 1 hour. The decapping reaction was stopped by TRIzol/chloroform extraction, and RNA was precipitated with an equal volume of isopropanol and 10ug glycogen, washed twice in 80% v/v ethanol, vacuum aspirated and dried at 42C for 5 min. RNA was quality controlled by NanoDrop to ensure no residual chaotropic salts (A230) or phenol (A270) remained. Decapped RNA heated to 65C for 5 min, and was circularized using 10U T4 RNA Ligase (NEB, #M0204) in 100ul 1x T4 DNA Ligase Buffer supplemented with 10% w/v PEG8000 and 10U Murine RNase Inhibitor at 16C for 24 hours. The circularization reaction was stopped by TRIzol/chloroform extraction and RNA was precipitated by isopropanol/ethanol and quality controlled as above.

Reverse transcription of specific mRNAs was performed using SuperScript IV (Invitrogen, #18090010) and gene-specific primer (S7 Table). Fifty nanograms of circularized RNA (cRNA) was denatured at 65C for 5 min in 14.25ul Solution 1 (9.5ul DEPC-treated ddH2O, 2ul 1uM reverse primer, 1ul 10mM dNTP mix, 1ul cRNA) with chilling on ice for 1 min. First strand synthesis was performed by addition of 5.75ul Solution 2 (4ul 5x SuperScript IV Buffer, 1ul 100mM DTT, 0.5ul 20U/ul Murine RNase Inhibitor, 0.25ul SuperScript IV) and incubation at 50C for 10 min. Reverse transcription reactions were stopped by heating to 80C for 10 min.

Specific PCR amplification of *gapdh* and *p28* sequences was conducted using Phusion polymerase (NEB) and gene specific primers (Supp Table 7), with products assessed by a 2% w/v agarose gel with 100bp molecular weight ladder (NEB).

Statistical Analyses

Statistical differences between *P. yoelii* wild-type and transgenic parasites were assessed via a two-tailed t-test on Graphpad Prism. Statistical differences between *P. falciparum* wild-type and PfCAF1ΔC parasites were assessed via a paired Wilcoxon test using R v. 3.3.1 [22] with p < 0.05 indicating statistical significance.

Data Availability Statement

*Proteomics Data*

Proteomics data have been deposited to the ProteomeXchange Consortium via the PRIDE partner repository with the dataset identifier PXD007042 [23].

Dataset Identifiers: SEL31: Wild-type parasites expressing GFPmut2 from a disrupted *p230p* locus (WT-GFP) replicate one, control for SEL32; SEL32: *ccr4-1^-^* experimental replicate one; SEL33: WT-GFP replicate two, control for SEL35; SEL34: WT-GFP replicate three, control for SEL36; SEL35 *ccr4-1^-^* experimental replicate two; SEL36: *ccr4-1^-^* experimental replicate three; SEL80: WT-GFP replicate one, control for SEL82; SEL82: *CAF1ΔC* Experimental replicate one; SEL83: WT-GFP replicate two, control for SEL85; SEL84: WT-GFP replicate three, control for SEL86; SEL85: *CAF1ΔC* Experimental replicate two; SEL86: *CAF1ΔC* Experimental replicate three. The mass spectrometry proteomics data have been deposited to the ProteomeXchange Consortium via the PRIDE partner repository with the dataset identifier PXD007042 [23].

Dataset Identifiers: SEL1: PY17XNL total proteomics, replicate one; SEL2: *ccr4-1^-^* total proteomics, replicate one.

*RNA Sequencing*

The DESeq2 output can be found in S2 Table and in the GEO depository (Accession number GSE101484).

RAW and processed transcriptomic files have been deposited at GEO (Accession # GSE101484)

**Extended Methods References**:

1. Mikolajczak SA, Aly AS, Dumpit RF, Vaughan AM, Kappe SH. An efficient strategy for gene targeting and phenotypic assessment in the Plasmodium yoelii rodent malaria model. Mol Biochem Parasitol. 2008;158(2):213-6. doi: 10.1016/j.molbiopara.2007.12.006. PubMed PMID: 18242728.

2. Jongco AM, Ting LM, Thathy V, Mota MM, Kim K. Improved transfection and new selectable markers for the rodent malaria parasite Plasmodium yoelii. Mol Biochem Parasitol. 2006;146(2):242-50. doi: 10.1016/j.molbiopara.2006.01.001. PubMed PMID: 16458371.

3. Balu B, Maher SP, Pance A, Chauhan C, Naumov AV, Andrews RM, et al. CCR4-associated factor 1 coordinates the expression of Plasmodium falciparum egress and invasion proteins. Eukaryotic cell. 2011;10(9):1257-63. doi: 10.1128/EC.05099-11. PubMed PMID: 21803864; PubMed Central PMCID: PMC3187058.

4. Methods in Malaria Research 2013. Available from: <https://www.beiresources.org/portals/2/MR4/Methods_In_Malaria_Research-6th_edition.pdf>.

5. Carter R. The Culture and Preparation of Gametocytes of Plasmodium falciparum for Immunochemical, Molecular, and Mosquito Infectivity Studies. In: Hyde JE, editor. Protocols in Molecular Parasitology. Methods in Molecular Biology1993. p. 67-88.

6. Miller JL, Harupa A, Kappe SH, Mikolajczak SA. Plasmodium yoelii macrophage migration inhibitory factor is necessary for efficient liver-stage development. Infect Immun. 2012;80(4):1399-407. doi: 10.1128/IAI.05861-11. PubMed PMID: 22252874; PubMed Central PMCID: PMCPMC3318411.

7. Lindner SE, Swearingen KE, Harupa A, Vaughan AM, Sinnis P, Moritz RL, et al. Total and putative surface proteomics of malaria parasite salivary gland sporozoites. Molecular & cellular proteomics : MCP. 2013;12(5):1127-43. doi: 10.1074/mcp.M112.024505. PubMed PMID: 23325771; PubMed Central PMCID: PMC3650326.

8. Reece SE, Ali E, Schneider P, Babiker HA. Stress, drugs and the evolution of reproductive restraint in malaria parasites. Proc Biol Sci. 2010;277(1697):3123-9. doi: 10.1098/rspb.2010.0564. PubMed PMID: 20484242; PubMed Central PMCID: PMCPMC2982055.

9. Mikolajczak SA, Vaughan AM, Kangwanrangsan N, Roobsoong W, Fishbaugher M, Yimamnuaychok N, et al. Plasmodium vivax liver stage development and hypnozoite persistence in human liver-chimeric mice. Cell Host Microbe. 2015;17(4):526-35. doi: 10.1016/j.chom.2015.02.011. PubMed PMID: 25800544; PubMed Central PMCID: PMCPMC5299596.

10. Deutsch EW, Mendoza L, Shteynberg D, Slagel J, Sun Z, Moritz RL. Trans-Proteomic Pipeline, a standardized data processing pipeline for large-scale reproducible proteomics informatics. Proteomics Clin Appl. 2015;9(7-8):745-54. doi: 10.1002/prca.201400164. PubMed PMID: 25631240; PubMed Central PMCID: PMCPMC4506239.

11. Kessner D, Chambers M, Burke R, Agus D, Mallick P. ProteoWizard: open source software for rapid proteomics tools development. Bioinformatics. 2008;24(21):2534-6. doi: 10.1093/bioinformatics/btn323. PubMed PMID: 18606607; PubMed Central PMCID: PMCPMC2732273.

12. Craig R, Beavis RC. TANDEM: matching proteins with tandem mass spectra. Bioinformatics. 2004;20(9):1466-7. doi: 10.1093/bioinformatics/bth092. PubMed PMID: 14976030.

13. Eng JK, Jahan TA, Hoopmann MR. Comet: an open-source MS/MS sequence database search tool. Proteomics. 2013;13(1):22-4. doi: 10.1002/pmic.201200439. PubMed PMID: 23148064.

14. Mellacheruvu D, Wright Z, Couzens AL, Lambert JP, St-Denis NA, Li T, et al. The CRAPome: a contaminant repository for affinity purification-mass spectrometry data. Nat Methods. 2013;10(8):730-6. doi: 10.1038/nmeth.2557. PubMed PMID: 23921808; PubMed Central PMCID: PMCPMC3773500.

15. Shteynberg D, Deutsch EW, Lam H, Eng JK, Sun Z, Tasman N, et al. iProphet: multi-level integrative analysis of shotgun proteomic data improves peptide and protein identification rates and error estimates. Mol Cell Proteomics. 2011;10(12):M111 007690. doi: 10.1074/mcp.M111.007690. PubMed PMID: 21876204; PubMed Central PMCID: PMCPMC3237071.

16. Choi H, Larsen B, Lin ZY, Breitkreutz A, Mellacheruvu D, Fermin D, et al. SAINT: probabilistic scoring of affinity purification-mass spectrometry data. Nat Methods. 2011;8(1):70-3. doi: 10.1038/nmeth.1541. PubMed PMID: 21131968; PubMed Central PMCID: PMCPMC3064265.

17. Munoz EE, Hart KJ, Walker MP, Kennedy MF, Shipley MM, Lindner SE. ALBA4 modulates its stage-specific interactions and specific mRNA fates during Plasmodium yoelii growth and transmission. Mol Microbiol. 2017. doi: 10.1111/mmi.13762. PubMed PMID: 28787542.

18. Smith RC, Vega-Rodriguez J, Jacobs-Lorena M. The Plasmodium bottleneck: malaria parasite losses in the mosquito vector. Mem Inst Oswaldo Cruz. 2014;109(5):644-61. PubMed PMID: 25185005; PubMed Central PMCID: PMCPMC4156458.

19. Lindner SE, Mikolajczak SA, Vaughan AM, Moon W, Joyce BR, Sullivan WJ, Jr., et al. Perturbations of Plasmodium Puf2 expression and RNA-seq of Puf2-deficient sporozoites reveal a critical role in maintaining RNA homeostasis and parasite transmissibility. Cell Microbiol. 2013;15(7):1266-83. doi: 10.1111/cmi.12116. PubMed PMID: 23356439; PubMed Central PMCID: PMCPMC3815636.

20. Anders S, Pyl PT, Huber W. HTSeq--a Python framework to work with high-throughput sequencing data. Bioinformatics. 2015;31(2):166-9. doi: 10.1093/bioinformatics/btu638. PubMed PMID: 25260700; PubMed Central PMCID: PMCPMC4287950.

21. Love MI, Huber W, Anders S. Moderated estimation of fold change and dispersion for RNA-seq data with DESeq2. Genome Biol. 2014;15(12):550. doi: 10.1186/s13059-014-0550-8. PubMed PMID: 25516281; PubMed Central PMCID: PMCPMC4302049.

22. Team RC. R: A language and environment for statistical computing.: R Foundation for Statistical Computing, Vienna, Austria. ; 2016.

23. Vizcaino JA, Csordas A, del-Toro N, Dianes JA, Griss J, Lavidas I, et al. 2016 update of the PRIDE database and its related tools. Nucleic Acids Res. 2016;44(D1):D447-56. doi: 10.1093/nar/gkv1145. PubMed PMID: 26527722; PubMed Central PMCID: PMCPMC4702828.
